# Germline soma communication mediated by gap junction proteins regulates epithelial morphogenesis

**DOI:** 10.1371/journal.pgen.1009685

**Published:** 2021-08-03

**Authors:** Aresh Sahu, Susnata Karmakar, Sudipta Halder, Gaurab Ghosh, Sayan Acharjee, Purbasa Dasgupta, Ritabrata Ghosh, Girish Deshpande, Mohit Prasad

**Affiliations:** 1 Department of Biological Sciences, Indian Institute of Science Education and Research-Kolkata, Mohanpur Campus, Mohanpur, Nadia, West Bengal, India; 2 Molecular and Behavioral Neuroscience Institute, University of Michigan Biomedical Science Research, Michigan, United States of America; 3 Department of Molecular Biology, Princeton University, Princeton, New Jersey, United States of America; New York University, UNITED STATES

## Abstract

Gap junction (GJ) proteins, the primary constituents of GJ channels, are conserved determinants of patterning. Canonically, a GJ channel, made up of two hemi-channels contributed by the neighboring cells, facilitates transport of metabolites/ions. Here we demonstrate the involvement of GJ proteins during cuboidal to squamous epithelial transition displayed by the anterior follicle cells (AFCs) from *Drosophila* ovaries. Somatically derived AFCs stretch and flatten when the adjacent germline cells start increasing in size. GJ proteins, Innexin2 (Inx2) and Innexin4 (Inx4), functioning in the AFCs and germline respectively, promote the shape transformation by modulating calcium levels in the AFCs. Our observations suggest that alterations in calcium flux potentiate STAT activity to influence actomyosin-based cytoskeleton, possibly resulting in disassembly of adherens junctions. Our data have uncovered sequential molecular events underlying the cuboidal to squamous shape transition and offer unique insight into how GJ proteins expressed in the neighboring cells contribute to morphogenetic processes.

## Author summary

Shape transitions between different subtypes of epithelial cells i.e., cuboidal, squamous and columnar are ubiquitous and are essential during organogenesis across animal kingdom. We demonstrate that heteromeric combination of gap junction proteins, *Drosophila* Innexin2 and *Drosophila* Innexin 4 (also known as Zero population growth or Zpg), expressed in the soma and germline of fly egg respectively, mediates the shape transition of cuboidal follicle cells to squamous fate. Interestingly, the two gap junction proteins likely participate as constituents of a calcium channel. Further, we show that somatic follicle cells and germline nurse cells communicate through calcium fluxes that activates STAT in the follicle cells. Activated STAT modulates the levels/ activity of junctional complexes thus aiding shape transition of cuboidal cells to squamous fate. These findings provide novel insights into how communication between different cell types with distinct origins achieve shape transitions essential for proper organ assemblies.

## Introduction

In the metazoans, several temporally coordinated interconnected morphogenetic events are instrumental for the transformation of a young embryo into an adult. Such events are spatiotemporally restricted and molecular processes underlying these events are causally as well as mechanistically linked. Among these, cell shape changes are especially crucial for the proper execution of several important developmental landmarks that lead to cell fate specification and patterning [1]. Thus, causes as well as consequences of cell shape changes, are being analyzed extensively in different biological contexts [2]. In this regard, epithelial morphogenesis has served as an extremely informative model.

Epithelial cells are classified into three basic categories based on the aspect ratio, namely cuboidal, squamous and columnar [3,4]. As the names indicate, cuboidal cells have similar height and width while the squamous cells are wider with reduced height. By contrast, columnar cells are less wide but of greater height. Depending on the individual cellular and/or developmental context, epithelial cells display distinct cell shape changes ranging from relatively simple ones like invagination, in-folding, and intercalation (convergent extension) to complex transformations including epiboly and branching morphogenesis [2]. One of the less explored aspects of epithelial morphogenesis is how the local intercellular interactions mediate these distinct shape changes during development. How these transitions are globally modulated, at a tissue level, to ultimately evolve a complex pattern is also relatively unclear. It is also apparent that in several instances different types of epithelial cells come in close proximity and interact with one another leading to distinct morphogenetic outcomes. For instance, close physical interactions between the columnar cells of wing imaginal disc proper with the overlying squamous peripodial cells plays an integral role in mediating wing morphogenesis in flies [5]. In fact, all organ assemblies including skin, lung, trachea, cervix and cornea are critically dependent on proper interactions and inter-convertibility between the epithelial subtypes [6–9].

In this regard, transformation of cuboidal cells into squamous cells is especially critical for aiding metazoan development [10]. This is important as squamous epithelial cells constitute a protective barrier around the tissues and organs, which helps to regulate the exchange of substances, ions, gases etc. between a given tissue and its environment [11]. Previous studies have indicated that most, if not all, of the shape transitions are modulated via the alterations in the intercellular adhesions between the neighboring cells [2]. Moreover, the cytoskeletal network plays an autonomous role in aiding the shape change by inducing alterations in adherens junctions [12,13]. Nonetheless, despite the functional significance, the precise mechanisms underlying acquisition of the squamous epithelial fate have remained elusive so far.

Here we have focused our attention on *Drosophila* ovary, which is an excellent model system to study cell shape transitions during development [14,15].Typically, a *Drosophila* ovary consists of approximately 100–150 oval structures or the egg chambers. An egg chamber is a reproductive unit of a female fly, which consists of 16 germline cells (cyst) enveloped by a layer of somatic follicle cells that are epithelial in nature and cuboidal in shape to begin with [15]. It develops through 14 stages, with the first seven stages being categorized as previtellogenic while the subsequent seven, as vitellogenic. As the oogenesis progresses, proliferating follicle cells accommodate the increasing size of the germline cells size till stage 6. Eventual expansion in the volume of the germline cells is accompanied by the shape change of the cuboidal follicle cells. At the anterior end, ~50 follicle cells of cuboidal fate referred to as anterior follicle cells (AFCs) in the text henceforth, reduce their lateral membrane (flatten) and expand their surface area (stretch) to cover the expanded germline cyst by acquiring squamous cell fate also denoted as stretched cell fate. In contrast, the posterior follicle cells elongate their lateral membranes enveloping the oocyte completely, thus procuring a columnar morphology. Despite the contrasting nature of the cell shape changes at the two termini, both these morphogenetic transformations assist in accommodating growth of the increasing size of the germ-line cyst [16].

Follicle cells, somatic in origin, are specified under the combined control of a number of signaling pathways including Notch, Transforming Growth Factor -beta, JAK-STAT, Epidermal Growth Factor and Hedgehog [17–22]. Furthermore, some of these signaling events also contribute to the generation of different epithelial subtypes. For instance, Notch signaling in the follicle cells modulates the adherens junctions remodeling to facilitate the follicle cell flattening [18]. In addition, the transcription factor, Hindsight presumably aids in remodeling adhesion between stretching AFCs [23]. While the Transforming Growth Factor signaling is thought to provide the temporal cue to initiate the cuboidal to squamous fate transition [17].

We recently demonstrated that Inx2, a gap junction protein mediates specification of a subset of follicle cells that are anteriorly localized [24]. Our results suggested that Inx2 controls the gradient of JAK-STAT signaling to ultimately influence Border Cell (BC) fate determination within the AFCs. Interestingly, AFCs, adjacent to the BC progenitors, are the ones that undergo cuboidal to squamous cell shape transition. Here we have investigated function of Inx2 in this shape transition during egg chamber morphogenesis. We report a series of molecular events initiated by the calcium flux passing through a heteromeric GJ channel at the germ-line/soma interface. Furthermore, we also demonstrate how these events may ultimately lead to a stereotypical cell shape change experienced by the AFCs. Taken together, our data have uncovered a novel molecular mechanism downstream of two GJ proteins that contributes to the cuboidal to squamous cell shape transition.

## Results

### Inx2 assists the cuboidal to squamous cell shape change observed in the AFCs

Graded activation of JAK-STAT signaling is essential for the acquisition of distinct follicle cell fates along the anterior-posterior axis of developing *Drosophila* egg chamber [19]. As we have demonstrated that gap junction protein Innexin2 (Inx2) potentiates the JAK-STAT signaling in the AFCs, we were curious to investigate the role of Inx2 in mediating the cell shape transition in developing follicle cells [24].

**Fig 1:**
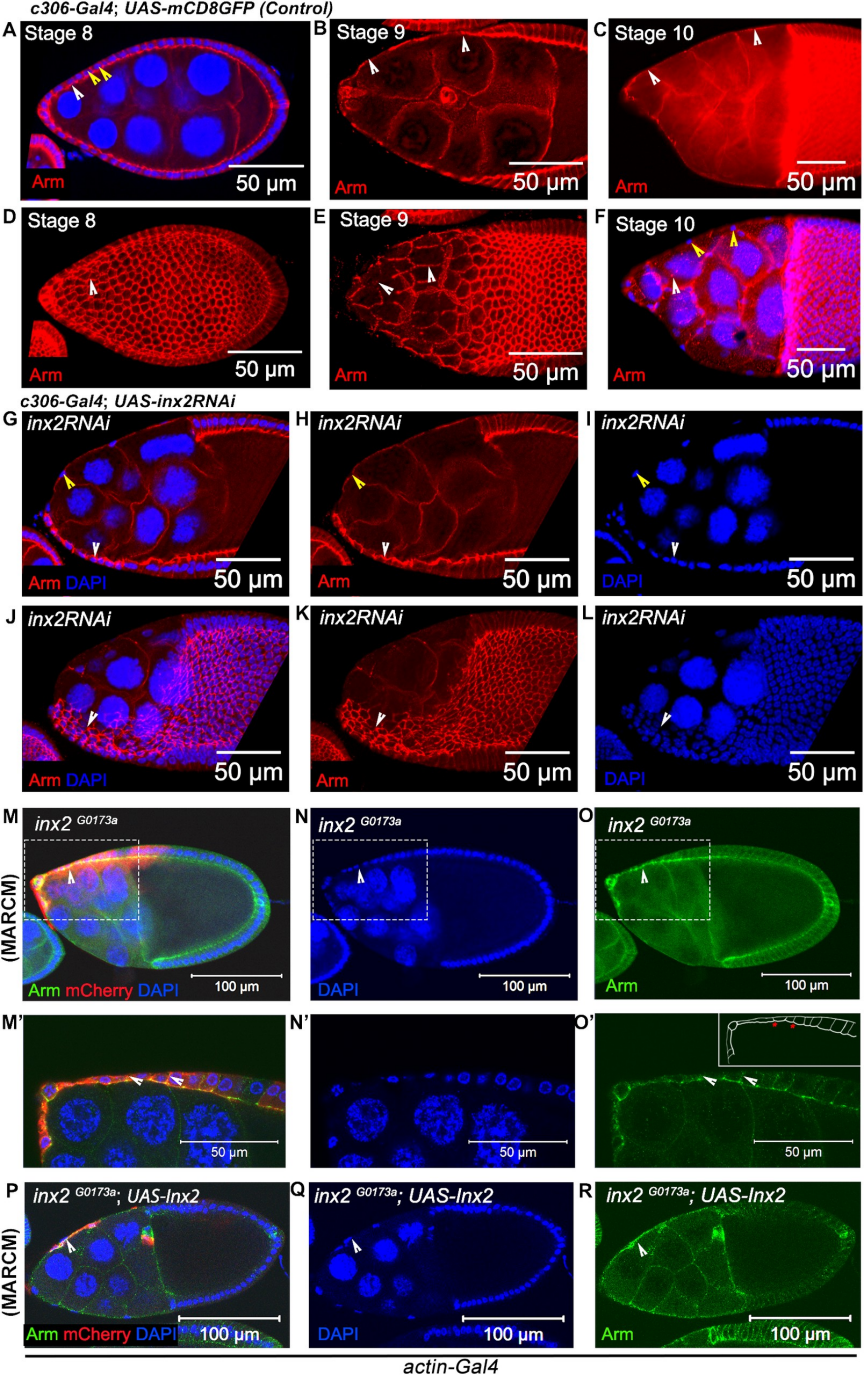
Inx2 mediates shape change in the AFCs. (**A-F)** Control Egg Chambers at indicated stages of oogenesis. Armadillo is shown in Red. White arrowhead marks the cuboidal cells in A., cells undergoing shape change in B. and squamous fate in C. Yellow arrow head in A. marks the closely space nuclei of cuboidal follicle cells (**A-C**) Sagittal sections of egg chambers exhibiting follicle cell shape change. (**D-F**) Surface views of the corresponding egg chambers depicted in A-C. **(G-I)** Stage 10 egg chambers of the indicated genotype exhibiting stretching defect. Armadillo and DAPI are imaged in Red and Blue respectively. White Arrowheads indicate cells that have failed to undergo stretching. Yellow arrow head marks the stretched cells. **(G)** Merged image of (H & I). (**J**) merged image of (K & L). **(M-R)** Stage 10 egg chambers of indicated genotypes with DAPI in Blue. Mosaic Analysis with a Repressible Cell Marker (MARCM) with *inx2*^*G0173a*^ allele. Armadillo is in Green. mCherry (Red) marks the *inx2*^*G0173a*^ mutant cells. **(M-O)** Arrowheads mark the *inx2*^*G0173a*^ mutant follicle cells that have failed to stretch. **(M’-O’)** The rectangular outlines in M-O are magnified as M’-O’ Arrowhead marks the lateral membrane. Inset in O’ is the schematic outline of the observed phenotype. Red Asterix mark the lateral membrane. **(P-R)** Note the rescue of stretching defect observed in *inx2*^*G0173a*^ mutant follicle cells by over expression of Inx2.

We thus decided to assess whether altering Inx2 function can influence the canonical cell shape changes AFCs undergo. In stage 8 egg chambers (*c306-Gal4; UAS*: *mCD8*: *GFP*) cuboidal AFCs display conspicuous, intercellular junctions with distinct lateral membranes and evenly distributed nuclei (Fig 1A). As oogenesis proceeds, the lateral membranes of the AFCs begin to shrink in size to initiate flattening coupled with significant expansion in their surface area (Fig 1A–1F). The surface area expansion is facilitated by the remodeling of the adherens junctions which eventually aids in the spreading of AFCs to acquire the squamous fate (Fig 1E) [18]. By stage 10, the squamous cells nuclei are spread out and intercellular junctions are inconspicuous due to considerably reduced lateral membrane (Fig 1C and 1F). To investigate the role of Inx2, we down regulated *Inx2* in the follicle cells using RNA interference approach. Since *c306-Gal4* expression spans the AFCs of stage 8 egg chambers, we employed it to overexpress *inx2RNAi* in the developing eggs (S1A–S1C Fig). Upon downregulation, 38.5 ± 1.2% of stage 10 egg chambers exhibited clustering of nuclei within the AFCs (Fig 1G–1L). This clustering of AFC nuclei in stage 10 egg chambers has been referred to as stretching defect henceforth in the text. It could arise either due to flattening defect where the shrinking of the lateral membrane is delayed or the follicle cells are unable to expand their surfaces **(**Figs 1G–1H and S1G–S1L). This suggests that Inx2 depletion affects the transitioning of cuboidal cells to squamous fate. Next, we tested if the RNAi construct indeed targets Inx2. We immunostained random overexpressing flip-out clones of *inx2RNAi* and observed lower levels of Inx2 protein compared to their wild-type counterparts suggesting that *inx2RNAi* indeed targets *Inx2* (S1D–S1F Fig). The presence of some residual Inx2 protein in the flip-out clones may be responsible for the modest penetrance. We also observed that smaller clones spanning the AFCs exhibited cell autonomous defects (S1G–S1I Fig). Curiously, in some instances, a few Inx2-depleted follicle cells were completely flattened however they did not exhibit the stretched morphology (S1J–S1L Fig). This particular phenotype suggested that the shape transition of the wild type AFCs likely proceeds via two stages, flattening being an intermediate stage that precedes stretching although we don’t understand the mechanistic underpinnings or nature of triggers that allow this progressive transition (S1J–S1L Fig).

To extend these observations, we performed clonal analysis using **M**osaic **A**nalysis with a **R**epressible **C**ell **M**arker (MARCM) with two different *Inx2* mutant alleles, *inx2*^*G0173a*^, and *inx2*^*G0059*^ [24]. MARCM technique allows labeling of homozygous mutant cells by activation of fluorescent reporter in an otherwise heterozygous genetic background of unlabeled cells. We analyzed homozygous mutant clones for both the alleles in developing egg chambers, which span over more than 7–8 follicle cells. Consistent with the earlier observations, homozygous mutant clones of *inx2* (*inx2*^*G0173a*^ and *inx2*^*G0059*^ alleles) in the AFCs exhibited defects in cuboidal to squamous cell shape transition in more than 20 egg chambers analyzed for each inx2 mutant (Figs 1M–1O and S2A–S2C). The mutant follicle cells were smaller with intact lateral membrane suggesting incomplete squamous cell morphogenesis (Fig 1M’ and 1O’). In addition, homozygous mutant follicle cell clones of *inx2*^*G0173a*^ or *inx2*^*G0059*^ exhibit severe down regulation of Inx2 protein (S2D–S2G Fig). Overall, our data suggest that Inx2 is involved in mediating the cuboidal to squamous transition of AFCs in developing egg chambers. The severity of defects is probably correlated with the extent of downregulation of Inx2 resulting in either severe flattening defects or moderate stretching defects.

Failure to expand the surface area resulted in inability of *inx2* mutant cells to cover nurse cells completely thus inhibiting the posterior movement of the main body follicle cells over the oocyte. As a result, the egg chambers with *inx2* mutant AFCs exhibited readily discernible morphological defects. (Fig 1M–1O). To assess if the effect of *inx2* on the cell shape change is spatially restricted and is confined to cuboidal to squamous cell shape transition, we also analyzed the *inx2* mutant clones in posterior follicle cells (PFCs), which acquire columnar shape in wild type egg chambers. Though *inx2* is also expressed in the PFCs, unlike the anteriorly localized clones, *inx2* clones spanning PFCs displayed normal cuboidal to columnar cell shape transition (S2B–S2C Fig). This observation suggests that Inx2 does not influence cuboidal to columnar shape transition observed in the PFCs. Thus, Inx2 activity is unlikely to be essential for the other cell shape changes during follicular morphogenesis. Furthermore, we did not observe any morphogenesis defects when the germline nurse cells were mutant for Inx2 (*inx2*^*G0173*^*)* further confirming a specific requirement of Inx2 in the AFCs. (S2H–S2J Fig)

To ascertain the specificity of the *inx*2 function, we overexpressed Inx2cDNA in the mutant cells using *actin-*Gal4 driver to assess if the shape transition defects observed in the *inx2*-depleted follicle cells are rescued. This experiment was done using MARCM analysis where *actin-*Gal4 is only active in follicle cells that are homozygous mutant for *inx2*. Satisfyingly, over-expression of *Inx2cDNA* in *inx2* mutant clones reversed the mutant phenotype completely as the mutant cells overexpressing Inx2 displayed normal flattening and stretching behaviors (Figs 1P–1R and S2K–S2M). It should be noted that we specifically considered mutant egg chambers where the inx2 mutant clones spanned at least 8 contiguous follicle cells (n ≥12). Together our data suggested that GJ protein Inx2 contributes to ovarian morphogenesis by regulating cuboidal to squamous cell shape transition seen specifically in the AFCs. It is important to note that overexpression of *inx2RNAi* with the centripetal-posterior follicle cell specific Gal4 (*c289b11-Gal4*) or main body follicle cell Gal4 (*cy2-Gal4*) didn’t affect the shape transition of AFCs (S2N–S2R Fig). Taken together these data suggest that Inx2 primarily functions in the AFCs that are undergoing cuboidal to squamous shape transition.

Next, we sought to assess if the fate of the follicle cells that do not undergo flattening was affected when *inx2* levels are compromised. Wild type follicle cells which transition from cuboidal to squamous fate, respond to Decapentaplegic (Dpp) signaling [17,25]. As Dpp signaling activates transcription of downstream target gene *dad (Daughters against Dpp*) [26,27], expression of *dad* reporter i.e., *dad-lacz* has been used as a readout of the Dpp signaling in the squamous follicle cells [17,28]. In wild type stage 8–9 egg chambers, *dad* expression initiates in the AFCs that would transition to squamous fate. In stage 10 egg chambers, *dad* expression is restricted to the squamous follicle cells (approximately 50 cells) that cover the nurse cells and the centripetal cells [29]. Dad expression is neither detected in the precursor cuboidal cells before stage 6 nor is it detected in the main body follicle cells that would envelope the growing oocyte (S3A Fig) [29]. We observed that the Inx2 depleted follicle cells that failed to acquire squamous shape, continue to express *dad*-*lacz* reporter (S3B–S3C Fig). This observation suggests that these follicle cells would have either stretched or are centripetal cells that would have migrated over the oocyte (S3A–S3C Fig). To validate this further, we employed another enhancer trap insertion, *[w*^*+*^ *lacz] BB-127*, that is specifically expressed in the centripetal cells and the nurse cells of egg chambers (S3D Fig) [30]. Consistent with our expectation, we observed that the Inx2 depleted follicle cells that failed to acquire squamous shape are devoid of *BB127-lacz* reporter expression. This confirmed that phenotypes induced by *inx2* loss are not due to the retention of the cuboidal fate nor are these cells constituents of main body follicle cells or the centripetal cells (S3E–S3F Fig). Moreover, we also detected the transcription factor, *eyes absent (eya)*, a marker for the AFCs even upon Inx2 depletion (S3G–S3I Fig). Altogether these results strongly suggest that Inx2-depletion specifically affects the AFCs that would have transformed to squamous FCs under normal circumstances.

Earlier reports have indicated possible involvement of Notch pathway components during the cell shape transition event under consideration. These studies also suggested that Notch signaling similarly influences the shape change in the AFCs without affecting their fate [18]. Thus, to examine if Inx2 functions via Notch to modulate the shape change in the AFCs, we analyzed the status of Notch signaling in the FCs compromised for *Inx2* (flip-out clones). We used two different Notch pathway readouts, Hindsight, a known target of Notch in the FCs, and NRE-GFP, a transcriptional reporter which is highly responsive to Notch activity [31]. Neither Hindsight nor the NRE-GFP levels appeared to significantly differ between the control and Inx2-depleted follicle cells suggesting that Inx2 likely modulates the shape transition independent of Notch (S3J–S3Q Fig).

Altogether these observations indicated that morphological defects associated with *inx2* depletion are not due to inappropriate specification of AFCs. Both the Notch and the BMP pathway targets are relatively unaffected in *inx2* depleted follicle cells which suggests that Inx2 may function independent of Dpp and Notch signaling to mediate shape change of the AFCs. Next, we were curious as to how the gap junction protein, Inx2 engineers the cuboidal to squamous cell shape transition in the AFCs.

### Inx2 mediated calcium flux plays a crucial role in cell shape transition

**Fig 2:**
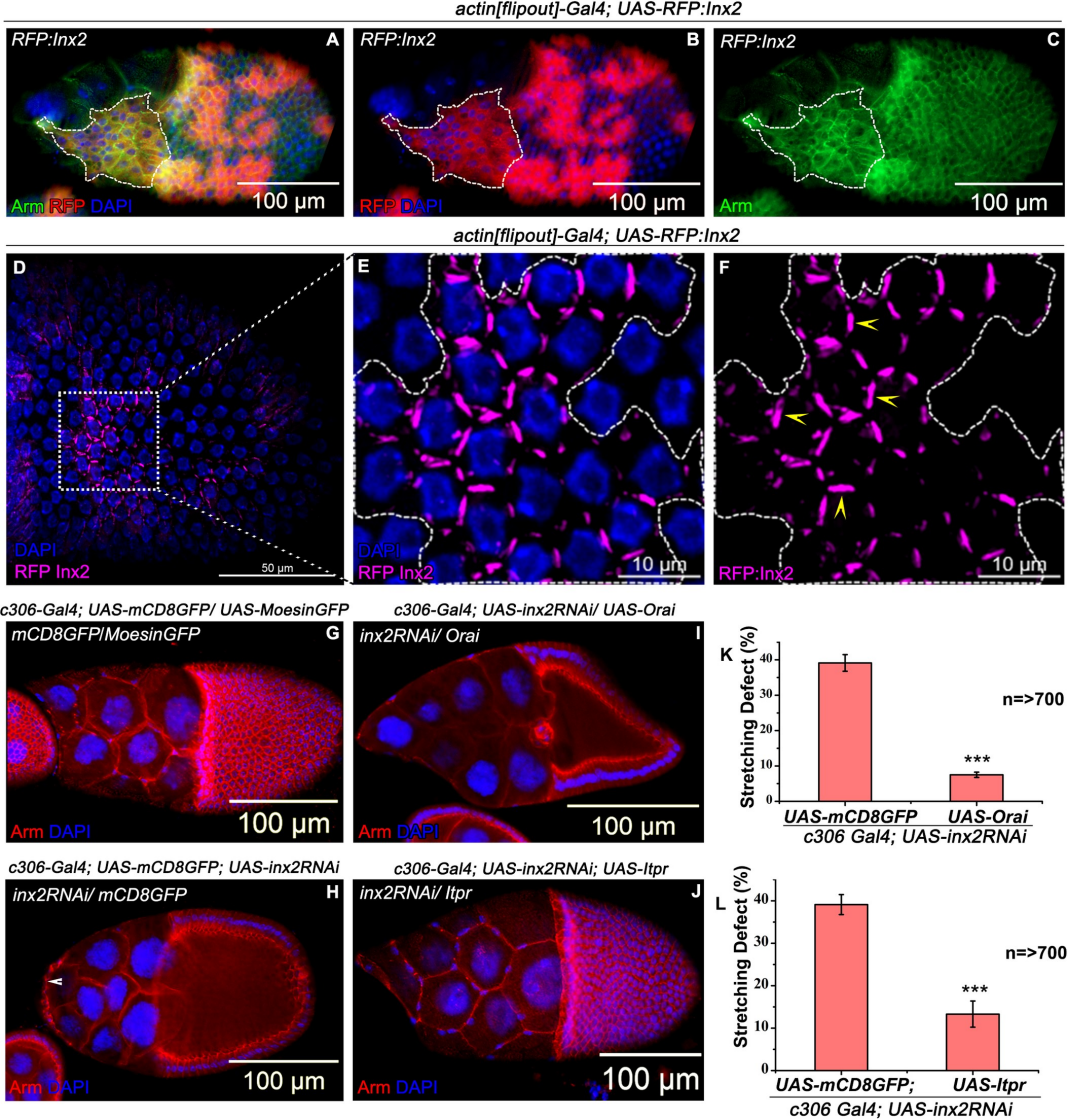
Channel activity of Inx2 affects shape change in AFCs. **(A-F)** Mosaic analysis-employing *actin [Flipout] Gal4* resulting in overexpression of UAS-RFP:Inx2. **(A-C)** Overexpression of RFP:Inx2 in anterior follicle cells exhibiting stretching defect. A is merge of B and C. The clones overexpressing RFP:Inx2 are marked in Red. Armadillo is in Green. Highlighted dotted lines mark the unstretched follicle cells. **(D-F)** Distinct localization of RFP:Inx2 in posterior follicle cells. RFP is in Magenta and DAPI in Blue. Boxed outline in the D is magnified in E and F. Highlighted dotted outline in E and F marks the RFP:Inx2 overexpressing clone in the posterior follicle cells. Yellow arrow heads in F marks the distinct membrane localization of RFP:Inx2 protein. **(G-L)** Rescue of stretching defect by elevating level of intracellular calcium in the follicle cells. **(K-L)** Quantification of the stretching defect in the indicated genotypes. **(G-I)** Over expression of Orai in Inx2-depleted follicle cells mitigates stretching defects. Armadillo in Red and DAPI in blue. Arrowhead in H marks the unstretched follicle cells. **(G-H and J)** Partial rescue of the Inx2-depleted follicle cell phenotype by over expression of Itpr. Armadillo is in Red and DAPI in Blue. n = number of egg chambers analyzed. *** indicates p-value <0.001. Error bars represent Standard Error of Mean.

Inx2 is a gap junction protein known to function as a channel in combination with other members of the same family. To examine if Inx2 may function as a component of a channel during epithelial shape transition, we employed a fusion transgene of Inx2 where coding portion of Inx2 has been tagged with RFP at the N-terminal end (*RFP*:*Inx2*) [32]. The resulting fusion protein (RFP:Inx2) is thought to function in a dominant negative manner that specifically affects the channel activity of Inx2 [32,33]. Using the flip-out technique, we overexpressed the RFP:Inx2 in a random subset of follicle cells. AFCs over-expressing *RFP*:*Inx2* construct failed to acquire squamous shape. (Fig 2A–2C). This phenotype resembled the follicle cell-stretching defect in Inx2 depleted AFCs (Fig 1) Moreover, the fusion protein, RFP:Inx2, was localized to the follicle cell membrane resembling the wild type Inx2 (Fig 2D–2F). Next, we assessed if over expression of RFP:Inx2 influences channel independent functions of Inx2 in developing eggs. Recently it has been reported that channel independent activity of Inx2 mediates the process of neolamination which involves fusion of the apical membranes of the border cell cluster with that of migrating centripetal cells in late egg chambers [34] To examine if the *RFP*: *Inx2* construct can affect neolamination, we independently expressed both *RFP*:*inx2* and *inx2RNAi* transgenes using the *slbo-Gal4 driver* in the border cells. Consistent with the previous report, expression of *inx2RNAi* induced neolamination in 48±0.63% of egg chambers. By contrast, overexpression of *RFP*:*Inx2* construct was unable to do so in 6.6%±2.3% egg chambers (S4A–S4D Fig). Altogether, these data suggest that over expression of *RFP*:*Inx2* doesn’t perturb the channel independent activity of Inx2 that is linked with neolamination and argue in favour of the possibility that the channel activity of Inx2 contributes to the shape transition of cuboidal to squamous epithelium.

We have previously shown that channel function of Inx2 mediates the passage of free calcium in somatic follicle cells, which plays a crucial role during specification of border cell fate [24]. Therefore, we were curious to check if Inx2 mediated calcium flux influences the cuboidal to squamous shape transition in the AFCs. As reducing Inx2 function quenches the calcium flux, we decided to test if simply increasing the level of calcium can rescue the defects induced by *inx2RNAi* expression in the follicle cells.

The level of calcium in the cytoplasm can be elevated either by allowing the extracellular calcium to enter the cells via store operated calcium channels or by releasing the calcium from the intracellular stores such as endoplasmic reticulum. Orai is a membrane associated store operated calcium channel that increases the intracellular level of calcium by increasing the influx of calcium from the extracellular milieu [35,36]. We overexpressed Orai in the Inx2-depleted AFCs and quantified the flattening defect. Depletion of Inx2 in AFCs exhibited flattening defect in 39±2.4% of egg chambers as compared to 7±0.8% observed in the Inx2 depleted AFCs that also overexpressed Orai (Fig 2G–2H and 2K). In addition, over expression of Inositol 1,4,5-tris-phosphate receptor (Itpr) also ameliorated the stretching defect in Inx2-depleted AFCs (13±3% versus 39±2.4% observed in control) (Fig 2I–2J and 2L). As Itpr mediates the release of calcium from intracellular stores, these data strongly argue that function of Inx2 during cell shape change is dependent on the availability of calcium. Furthermore, its ability to function as a calcium channel is likely critical to mediate the cell shape changes.

### Inx2 functions via JAK-STAT signaling to regulate follicle cell stretching

**Fig 3:**
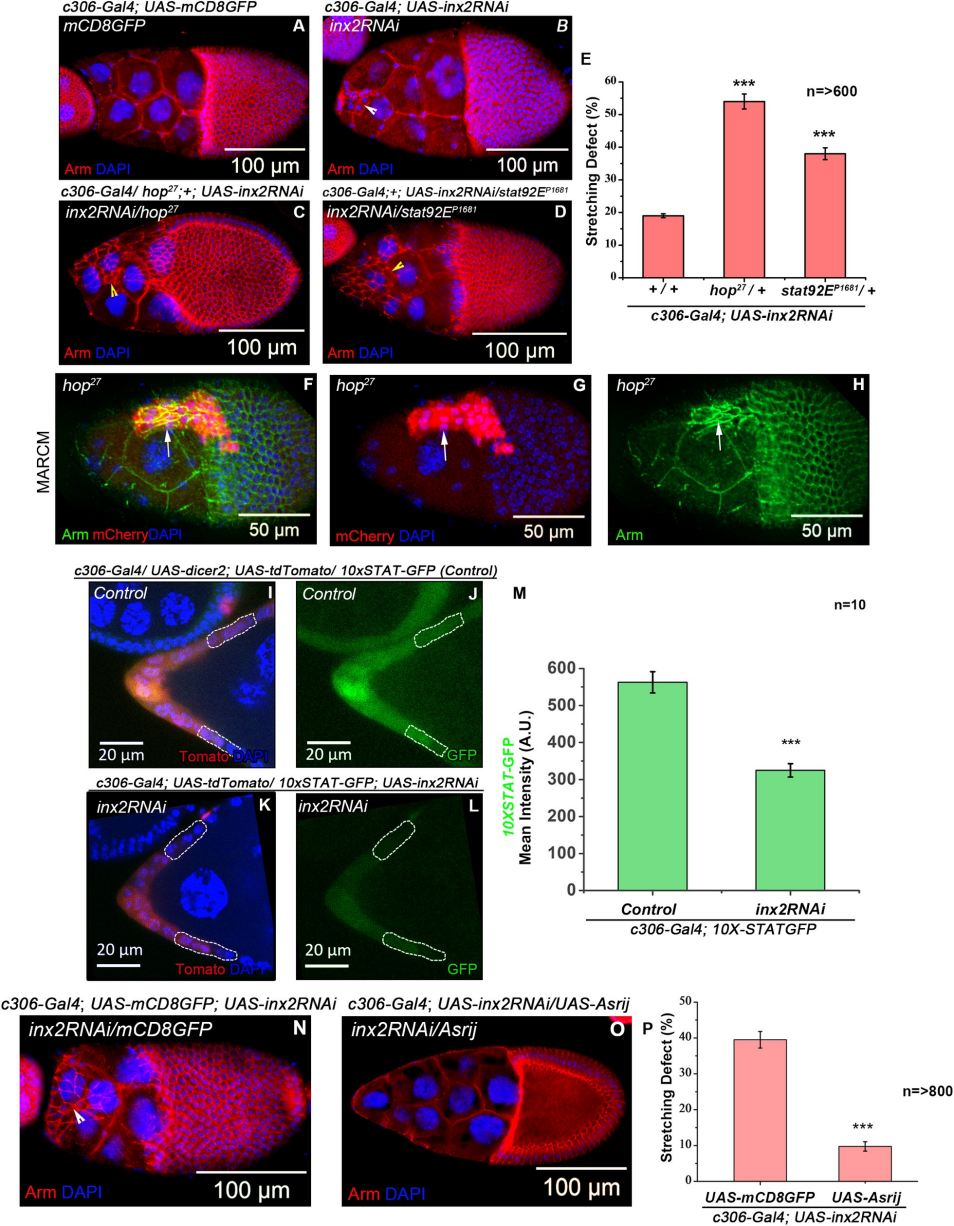
Inx2 functions through JAK-STAT pathway to modulate the shape change in the AFCs. **(A-D)** Stage 10 egg chambers of indicated genotypes stained with anti-Armadillo in Red and DAPI in blue. Arrowheads mark the unstretched follicle cells. **(E)** Quantification of the stretching defect of the indicated genotypes. Please note the increase in stretching defect exhibited by Inx2-depleted follicle cells in *hop*^*27*^*/+* and *stat92E*^*P1681*^*/+* genetic background. **(F-H)** MARCM Analysis. *hop*^*27*^ mutant follicle cells marked by expression of mcherry in Red, Armadillo in Green and DAPI in Blue. Please note the unstretched follicle cells marked by arrow. **(I-M)** Stage 8 egg chamber of indicated genotypes. White dotted line outlines the AFCs that would stretch to acquire squamous fate. *10XSTAT*-GFP expression is in Green, Td Tomato in Red and DAPI in blue. **(M)** Quantification of the *10XSTAT*-GFP of control *(c306-Gal4/ UAS-dicer2; 10XSTAT-GFP/UAS-tfTomato) and inx2RNAi (c306-Gal4; 10XSTAT-GFP/ UAS-tdTomato; UAS-inx2RNAi)***. (N-P)** Rescue of stretching defect of Inx2-depleted follicle cells by over expression of Asrij. **(N-O)** Stage 10 egg chambers of indicated genotypes stained with anti-Armadillo in Red and DAPI in blue. Arrowhead marks the unstretched follicle cells. **(P)** Quantification of the stretching defect in the indicated genotypes. Please note the rescue in the stretching defect when Asrij is overexpressed in the Inx2-depleted follicle cells. *** represents p-value <0.001. n stands for the number of egg chamber analyzed. Error bars represent Standard Error of Mean.

As calcium influx seems to play an important role during the process, we decided to investigate how Inx2 mediated calcium flux modulates the particular shape transition. We have previously shown that Inx2 mediates STAT activity to influence BC specification [24]. We thus wondered if Inx2 functions through the JAK-STAT pathway in this context. To test if the components of the JAK-STAT pathway interact genetically with *inx2* in the AFCs undergoing flattening and stretching, we down regulated Inx2 function in genetic backgrounds where JAK-STAT pathway was compromised. To this end, we used flies heterozygous for *hopscotch* (*hop*^*27*^: null allele of *Drosophila* JAK) and *stat (stat92E*^*P1681*^: a null allele of *Drosophila* Stat92E). We quantified the stretching defects displayed by the AFCs that were simultaneously compromised for *inx2* and JAK-STAT signaling. This experiment was done at 25°C to specifically induce a modest phenotype by *inx2RNAi* overexpression. We have employed this experimental condition as a ‘sensitized background’ to assess if JAK-STAT pathway components can modify (either suppress or enhance) the phenotypes induced by Inx2RNAi. Compared to the 19 ± 0.6% stretching defect exhibited by the Inx2 depleted AFCs (*c306-Gal4; UAS-inx2RNAi)*, we observed 53% ± 2.3% stretching defect in *hop*^*27*^ heterozygous (*c306-Gal4/ hop*^*27*^*; UAS-inx2RNAi/+*) and 37 ± 1.9% stretching defect in *stat92E*^*P1681*^ heterozygous (*c306-Gal4/+; UAS-inx2RNAi/stat92E*^*P1681*^) background at 25°C (Fig 3A–3E). This result shows that simultaneously compromising JAK-STAT pathway components can enhance the stretching defects induced by down regulation of Inx2. These data raised the possibility that Inx2 may influence the JAK-STAT pathway to achieve the cell shape change in the AFCs.

Next, we were curious if the JAK-STAT signaling *per se* affects the shape change of AFCs. Mutant AFCs clones of *hop*^*27*^ or *stat92E*^*(P1681)*^ retained higher levels of adherens junction protein Armadillo and failed to stretch like the Inx2 mutants (Figs 3F–3H and S5A–S5C). This suggests that depletion of JAK-STAT signaling can inhibit cuboidal to squamous transition of AFCs.

We also tested the status of JAK-STAT signaling in Inx2 depleted AFCs in early stage 8 egg chambers prior to cell shape transition. To monitor the extent of JAK-STAT pathway activity in AFCs, we employed the *10XSTAT-GFP* reporter. The extent of STAT activation correlates with the intensity of GFP-specific signal [37]. As previously reported, in control egg chambers (*10XSTAT-GFP/+*) a decreasing gradient of the STAT reporter was observed from the terminal anterior end to the center of the egg chamber (Fig 3I–3J) [24]. Here we subsequently quantified and compared the GFP intensity (as a proxy of STAT activity) in both, the control and the Inx2 depleted AFCs that would have presumably acquired the squamous fate. To do this, we evaluated the GFP intensity in the AFCs that are 3 cells away from the anterior polar cells to avoid the border cells. In control, the average reporter intensity (STAT activity) was observed as 562.78 ± 28.69 a.u., while Inx2 depleted AFCs exhibited lower STAT activity with average reporter intensity of 324.74±18.14 a.u. (Fig 3I–3M). Thus, down regulation of Inx2 decreases the activity of JAK-STAT signaling in the AFCs.

If the ability of Inx2 to directly modulate the JAK-STAT pathway is crucial for the specific shape transition, elevating the JAK-STAT signaling in Inx2 depleted follicle cells should ameliorate the flattening defect. Interestingly, simple over expression of STAT in the Inx2-depleted AFCs didn’t rescue the stretching defects (S5D–S5F Fig). Next, we tested if the increasing the activity of STAT can rescue this shape transition defect. However, co-expression of constitutively active JAK (*UAS-Hop*^*Tum*^) and *inx2RNAi* in the AFCs resulted in lethality.

**Fig 4:**
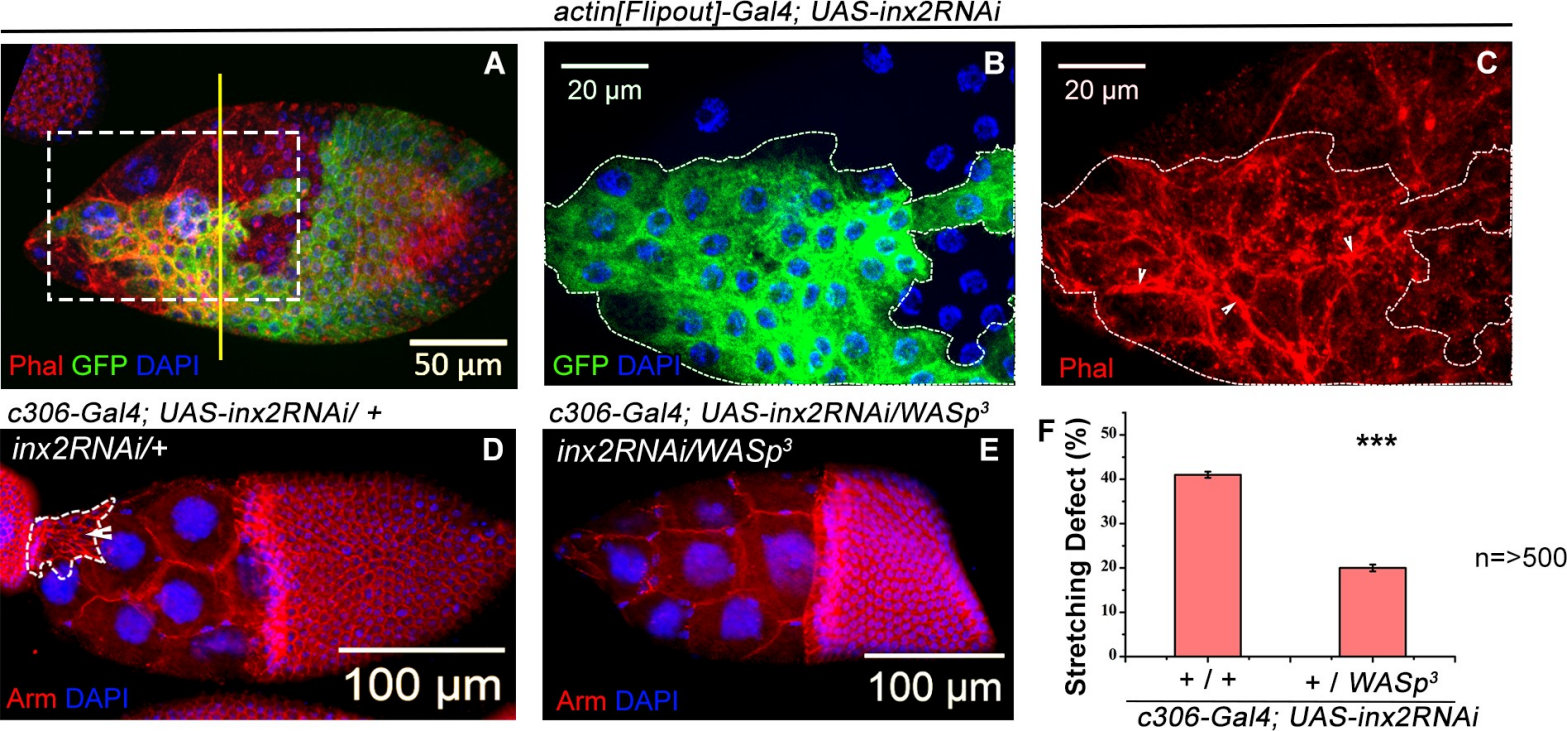
Inx2 modulates Wasp to effect shape change of AFCs. **(A)** Mosaic analysis-employing *actin[Flipout]-Gal4* resulting in overexpression of *inx2RNAi* in clones. White box marks the region magnified in B and C. **(B)** White dotted line denotes clones over expressing *inx2RNAi* is depicted in Green (GFP). **(C)** Phalloidin imaged in Red indicates the status of F-actin in the *inx2RNAi* over expressing clone. Note the presence of elevated levels of F-actin in the Inx2-depleted follicle cells. Yellow line demarcates the anterior follicle cells that will stretch from the centripetal and main body follicle cells. **(D-E)** Stage-10 egg chambers of indicated genotypes. Anti-Armadillo shown in Red and DAPI is in blue. White dotted line outlines the unstretched follicle cells. **(F)** Quantification of stretching defects seen in the samples from the indicated genotypes. Please note the rescue of the Inx2-depleted stretching defect in *WASp*^*3*^*/+* background. *** represents p-value <0.001. n = number of egg chamber analyzed. Error bars represent Standard Error of Mean.

To circumvent this problem, we sought to use an indirect approach to elevate STAT activity. Asrij/OCIAD1 (ovarian carcinoma immune-reactive antigen (OCIA) domain family) is an endosomal protein that promotes STAT phosphorylation both in embryonic stem cells and *Drosophila* hematopoietic stem cells [38]. Supporting the conclusion that Asrij can potentiate STAT activity in follicle cells, we confirmed that, overexpression of Asrij in the wild type follicle cells increased the levels of *10XSTAT-GFP* (S5G–S5K Fig). We subsequently co-expressed *Drosophila* Asrij in the Inx2 compromised follicle cells. Satisfyingly overexpression of Asrij in the Inx2 depleted follicle cells rescued the stretching defect (39.5±2.3% to 9.8±1.3%) (Fig 4N–4P). These data suggested that Inx2 may regulate STAT activity to mediate epithelial cell shape transition in the AFCs. Rescue of stretching defects in the Inx2 depleted AFCs by over expression of endosomal protein Asrij suggested that STAT activity could be one of the mediators of shape change of AFC. However, we cannot rule out the contribution of other mediators or signaling cascades affected by the changes in Asrij levels/activity.

### Inx2 modulates myosin through WASp to facilitate follicle cell shape transition

In a number of developmental contexts, cell shape changes depend on alterations in actin/myosin cytoskeleton. We decided to test if similar cytoskeletal changes accompany cuboidal to squamous cell shape transition in the AFCs. We observed higher levels of F-actin in the Inx2 depleted follicle cells (Fig 4A–4C). In a gastrulating embryo, JAK-STAT signaling represses actin nucleator, WASp to spatially regulate myosin activity to facilitate apical constriction [39]. As STAT activity is downstream of Inx2 in this context, we wondered if Inx2 suppresses actin nucleator WASp to influence actin levels. To test this, we compared cell-stretching defects in Inx2-depleted follicle cells (*c306-Gal4; UAS-inx2RNAi)* in the wild type and *WASp*^*3*^ heterozygous background respectively. If Inx2 employs *WASp* repression to mediate follicle cell stretching, reducing WASp level in Inx2 deficient background should mitigate follicle cell flattening defect. Consistently, we observed quantifiable improvement in the stretching defects. Inx2 depleted follicle cells in *WASp*^*3*^ heterozygous background exhibited 19±0.7% stretching defect compared to 43±0.7% stretching defect to that observed in wild type background at 29°C (Fig 4D–4F). This result suggested that Inx2 represses WASp activity to assist the flattening of the AFCs as the oogenesis progresses.

**Fig 5:**
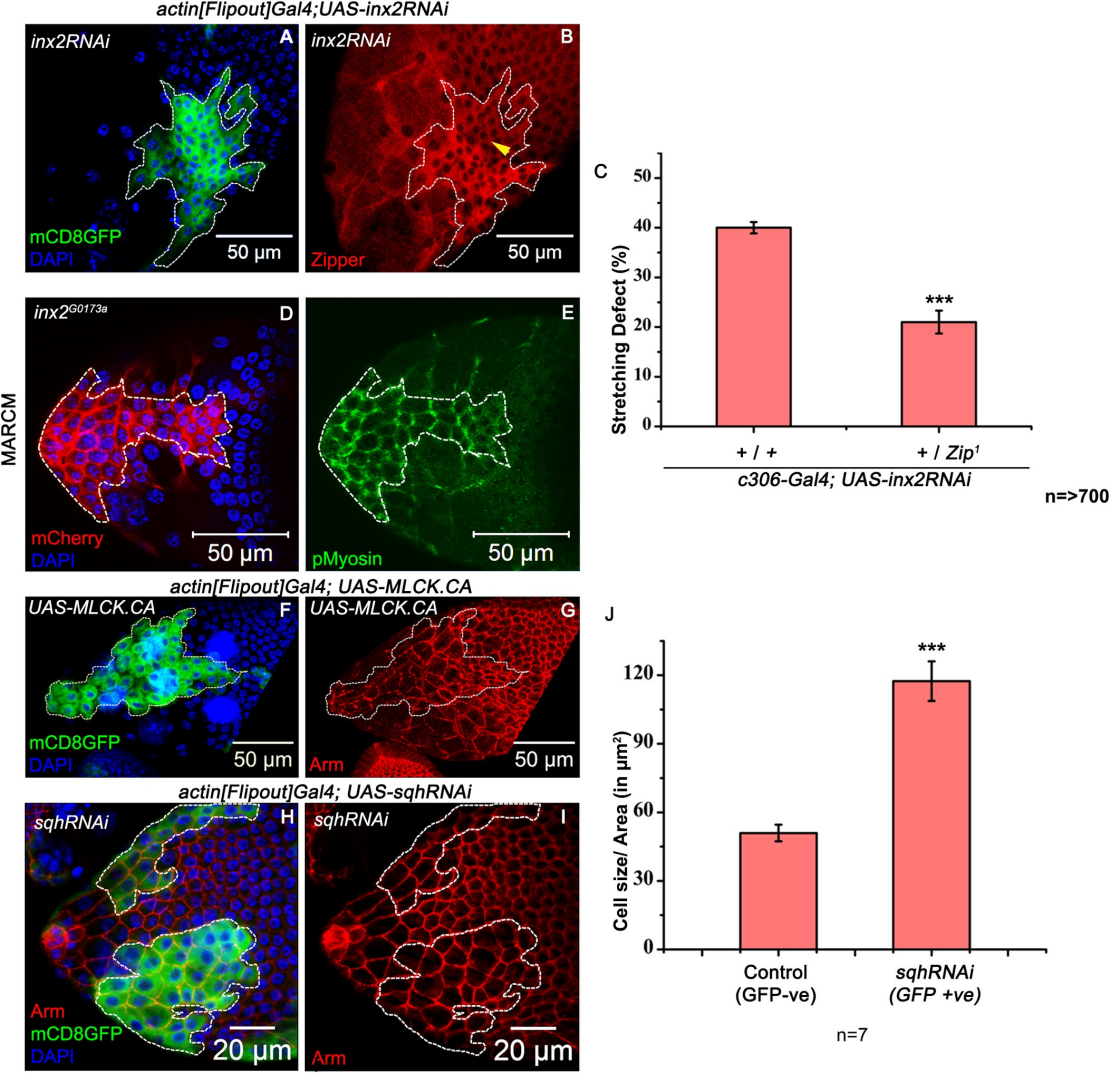
Inx2 influences myosin activity to execute the shape change. **(A-B)** Mosaic analysis-employing *actin[Flipout]-Gal4* resulting in overexpression of *inx2RNAi* in clones. White dotted outline marks the clone expressing GFP in Green, DAPI in Blue. Anti-Zipper staining is imaged in Red. Elevated levels of Zipper in B. are observed in Inx2-depleted follicle cells compared to the control neighbouring cells undergoing shape change. Yellow arrow head marks the adjacent main body follicle cells of similar size as that of unstretched AFCs. Please note Zipper enriched only in the unstretched AFCs. **(C)** Quantification of stretching defects for indicated genotypes. Please note the rescue in the stretching defect of the Inx2-depleted follicle cells in *zip1/+* background. *** represents p-value <0.001. n stands for the number of egg chamber analyzed. **(D-E)** MARCM analysis with *inx2*^*G0173a*^ allele. mCherry imaged in Red marks the *inx2*^*G0173a*^ mutant clone depicted in white outline. Phospho-myosin is shown in Green. Please note the elevated levels of Phospho-myosin in unstretched AFCs. **(F-G)** Clonal over expression of constitutively active myosin light chain kinase in the follicle cells **(F)** Clone is marked in Green (GFP) and highlighted by dotted outline. **(G)** Anti-Armadillo in Red. Please note that excess myosin activity causes stretching defect in the AFCs. **(H-I)** Clonal over expression of *sqhRNAi* mediated by *actin [Flipout] Gal4*. Clones are marked in Green and highlighted with a dotted outline. Armadillo in Red and DAPI in Blue. **(J)** Quantification of the follicle cell area. n = number of follicle cells analyzed. Please note increase in cell area where the Sqh function is down regulated. *** represents p-value <0.001. Error bars represent Standard Error of Mean.

To better understand the cytoskeletal changes, we compared the distribution of Zipper i.e., Myosin heavy chain between the wild type and Inx2-depleted follicle cells (Flip out overexpressing clones). In the wild type egg chambers, a basal level of Zipper was observed in the cuboidal follicle cells (S6A–S6B Fig). While, in the Inx2-depleted follicle cells, we observed higher levels of Zipper staining than the stretched follicle cells, (Figs 5A–5B and S6A–S6B). Similar observation was made for STAT depleted AFCs (S6C–S6E Fig). Moreover, we also observed that the stretching defect of the Inx2-depleted follicle cells (*c306-Gal4; UAS-inx2RNAi)*, was mitigated in *zip*^*1*^ (null allele) heterozygous background at 29°C (21±2.3% in *zip*^*1*^*/+* v/s 40±1.9% in wild type background) (Fig 5C). Similar rescue in the stretching defect of STAT depleted follicle cell was observed in *zip*^*1*^ heterozygous background (3.86±1.16% in *zip*^*1*^*/+ vs* 88.35±1.36% in wildtype background) (S6F–S6H Fig). Together these data suggest that the function of Inx2 and STAT affects the actin/myosin cytoskeleton to facilitate the stretching observed in the AFCs.

In *Drosophila*, non-muscle myosin activity depends upon the phosphorylation of its regulatory component, myosin regulatory light chain (MRLC) at threonine-20 and serine-21 amino acid residue [40]. To assess if the myosin activity was different between the wild type and Inx2-depleted follicle cells, we stained egg chambers with anti-pMyosin (phospho-myosin regulatory light chain) antibody, which specifically detects the phosphorylated form of myosin regulatory light chain [41]. In control egg chambers, the cuboidal epithelial cells display lower levels of junctional phospho-myosin (S6I–S6J Fig). As the oogenesis progressed and the AFCs initiated stretching, loss of phospho-myosin was observed from the junctions. In the Inx2 mutant follicle cells, we observed a high level of phospho-myosin accumulation in the membrane and the junctions of the unstretched follicle cells (Fig 5D–5E). This suggests that Inx2 likely affects both the activity and levels of myosin in *Drosophila* follicle cells that are undergoing cuboidal to squamous fate transition, and loss of Inx2 leads to inappropriate stabilization of Myosin levels/activity. Similar accumulation of phospho-myosin was observed in STAT depleted AFCs (S6K–S6N Fig).

To further validate that the higher activity of myosin can contribute to the cell shape transition, we overexpressed a constitutively active form of Myosin Light Chain Kinase (MLCK) in random subset of AFCs using the flip-out technique. MLCK phosphorylates the myosin regulatory light chain and is thought to increase the non-muscle myosin activity [42]. We observed that follicle cells expressing constitutively active MLCK exhibit a delay in the stretching process, which also correlated with the retention of their adherens junction component DE-Cadherin (Fig 5F–5G) (see below for further details).

Reciprocally, to assess if temporally coordinated reduction in myosin activity can promote follicle cell stretching, we down regulated the function of regulatory light chain of the non-muscle type 2 myosin encoded by *spaghetti squash* (*sqh*) in the AFCs by RNA interference. Interestingly, we observed that follicle cells over expressing *sqhRNAi (Flip out clones)* exhibited larger surface area (117.4±8.6 μm^2^) compared to the nearby wild type cells (51±3.6 μm^2^) (Fig 5H–5J). This observation suggested that precocious reduction in myosin activity can promote (and hasten) stretching of the AFCs. Altogether our data suggest that Inx2 function attenuates myosin activity to facilitate the flattening of AFCs.

### Inx2 permits adherens junction disassembly

Myosin accumulation affects the disassembly of adherens junction (AJ) and has been shown to be crucial for modulating cell shape changes in epithelial cells [18,43]. Intriguingly, Inx2 depleted unstretched follicle cells also exhibit accumulation of myosin at their cell junctions. Furthermore, follicle cells expressing constitutively active MLCK also displayed retention of Armadillo, a component of adherens junction complex (Fig 5F–5G). We thus wondered if Inx2 permits the adherens junction disassembly via myosin distribution in the AFCs that are undergoing shape transition.

**Fig 6:**
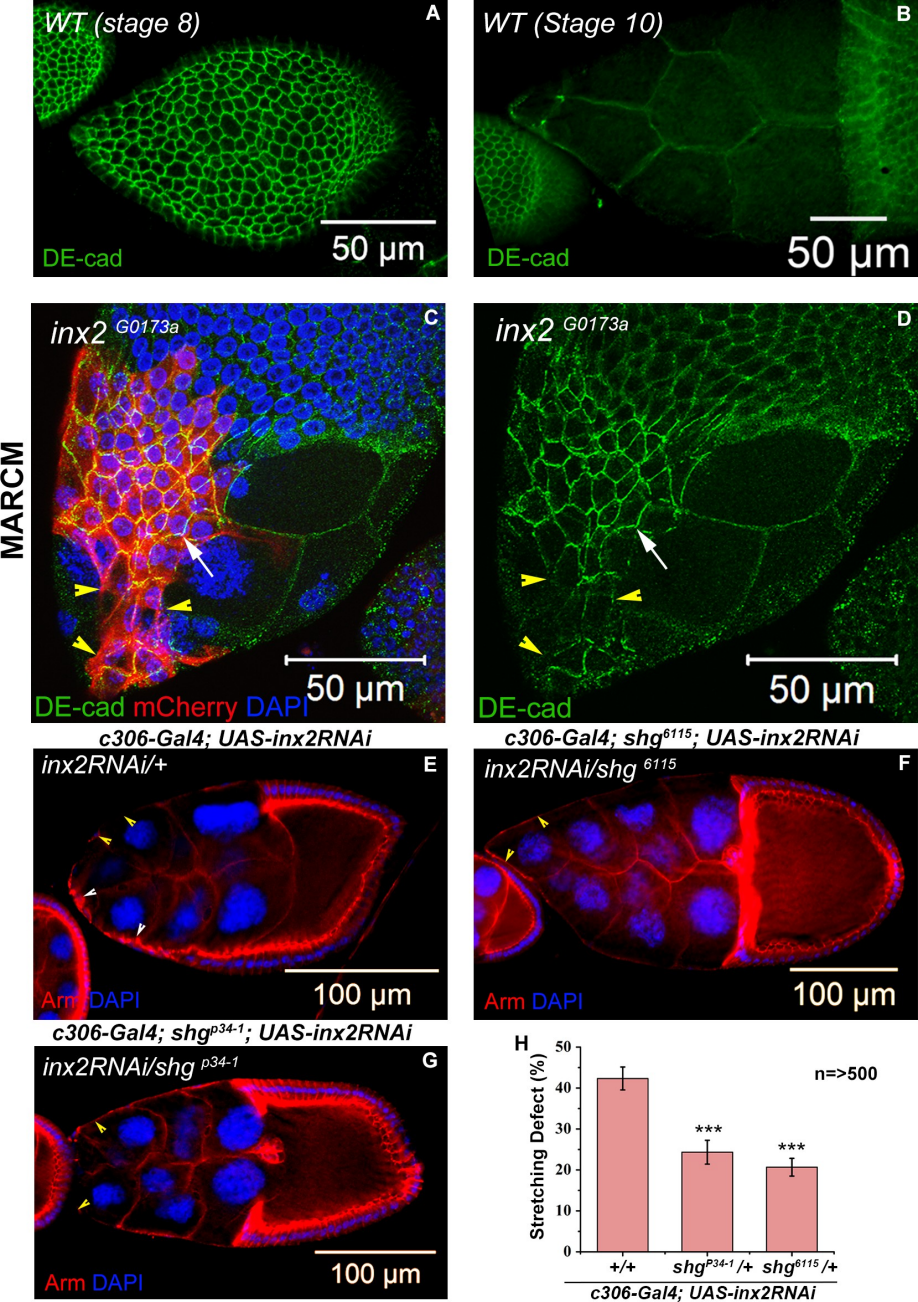
Inx2 modulates shape transition possibly via changes in the levels of DE-Cadherin. **(A-B)** *Wild type* (*WT*) egg chambers stained with anti-DE-Cadherin shown in Green. Note that unlike the squamous cells in B, DE-Cadherin is enriched in the cuboidal follicle cells in A. **(C-D)** MARCM analysis. Stage 10 egg chambers carrying clones of *inx2*^*G0173a*^ mutant follicle cells, which are marked with mCherry in Red. Note the retention of E-Cadherin in the unstretched follicle cells (Arrow). Yellow arrow head marks the interface of clone and nonclone boundary where DE-Cadherin is downregulated. **(E-H)** Partial rescue of stretching defect is seen in *shotgun/ DE-cadherin* heterozygous background. **(E-G)** Stage 10 egg chambers of indicated genotypes stained with Anti-Armadillo in Red and DAPI in Blue. White Arrowhead in E marks the unstretched AFCs. Yellow Arrowheads mark the stretched follicle cells. **(H)** Quantification of stretching defect in different genetic backgrounds. n = number of egg chambers analyzed. Note the rescue in stretching defect of the Inx2-depleted follicle cells in *shotgun* heterozygous background. *** represents p-value <0.001. Error bars represent Standard Error of Mean.

To check this, we examined the status of adherens junction component *Drosophila* Epithelial-Cadherin (DE-Cadherin) in the *inx2* mutant follicle cells. We stained egg chambers with anti-DE-Cadherin antibody, a major component of adherens junctions [44]. In wild type stage 8 egg chambers, we observed distinct, continuous DE-Cadherin localization at the junctions of the cuboidal follicle cells (Fig 6A). By contrast, the DE-Cadherin accumulation appeared to be discontinuous with several gaps at the interface of clone- non clone follicle cells in late stage 8 or early stage 9 egg chambers (yellow arrow heads in Fig 6D). Upon completion of stretching, the DE-Cadherin specific signal was nearly absent in the AFCs suggesting that disassembly of adherens junction components is concomitant with the flattening process (Fig 6B). In contrast, the Inx2 mutant follicle cells in stage 10 egg chambers continued to exhibit high levels of DE-Cadherin suggesting that disassembly and the removal of DE-Cadherin was affected in Inx2 mutants (Fig 6C–6D).

To examine if the retention of DE-Cadherin contributes to the follicle cell stretching defect observed in Inx2 depleted egg chambers (*c306-Gal4; UAS-inx2RNAi)*, we down-regulated Inx2 function in wild type and in Shotgun (Shg) (*Drosophila* cadherin; *shg*^*p34-1*^, *shg*^*6114*^) mutant background at 29°C. Indeed, stretching defects induced by the loss of inx2 were partially rescued by reduction in DE-Cadherin levels/activity from 42.3±2.8% in wild type background to 24.3±2.9% and 20.6±2.2% in *shg*^*p34-1*^ and *shg*^*6115*^ heterozygous background respectively (Fig 6E–6H). Inx2 thus regulates the levels of DE-Cadherin in the AFCs to enable follicle cell stretching.

Altogether our data suggests that Inx2 mediates JAK STAT signaling to mediate the flattening of the AFCs in the *Drosophila* egg chambers. Our genetic and cell biological analysis argues that Inx2 represses *WASp* to modulate myosin activity critical for the disassembly of adherens junction components during flattening of AFCs. These data are consistent with earlier reports, which documented a similar influence of STAT on the cell shape changes in a different cell biological context [39].

### Germline specific GJ protein Inx4 (Zpg) influences the AFCs similarly as Inx2

As increase in the size of the germline cells coincides with the shape transition in the follicular epithelium, we were curious to know if the nurse cells participate in the flattening of the follicle cells [45,46]. A possible mode of germline-soma communication in this context could be via formation of a gap junction channel.

Inx2 is a gap junction component and a member of a calcium channel family. Typically, proteins of this family work in combination to form a hetero-dimeric or a multi-component channel(s) that facilitate transport of small molecules including ions or metabolites. Of note, Inx2 has been shown to genetically interact with Inx4 (hereafter referred to as Zpg), a germline-specific gap junction protein to assist the formation of cyst and egg chamber [47]. Importantly, nurse cell specific expression domain of Zpg seems to be in close proximity of the follicle cell specific expression domain of Inx2 [48]. Based on these two observations we wondered if Inx2 from the follicle cells works in conjunction with Zpg at the FC-NC junction.

**Fig 7:**
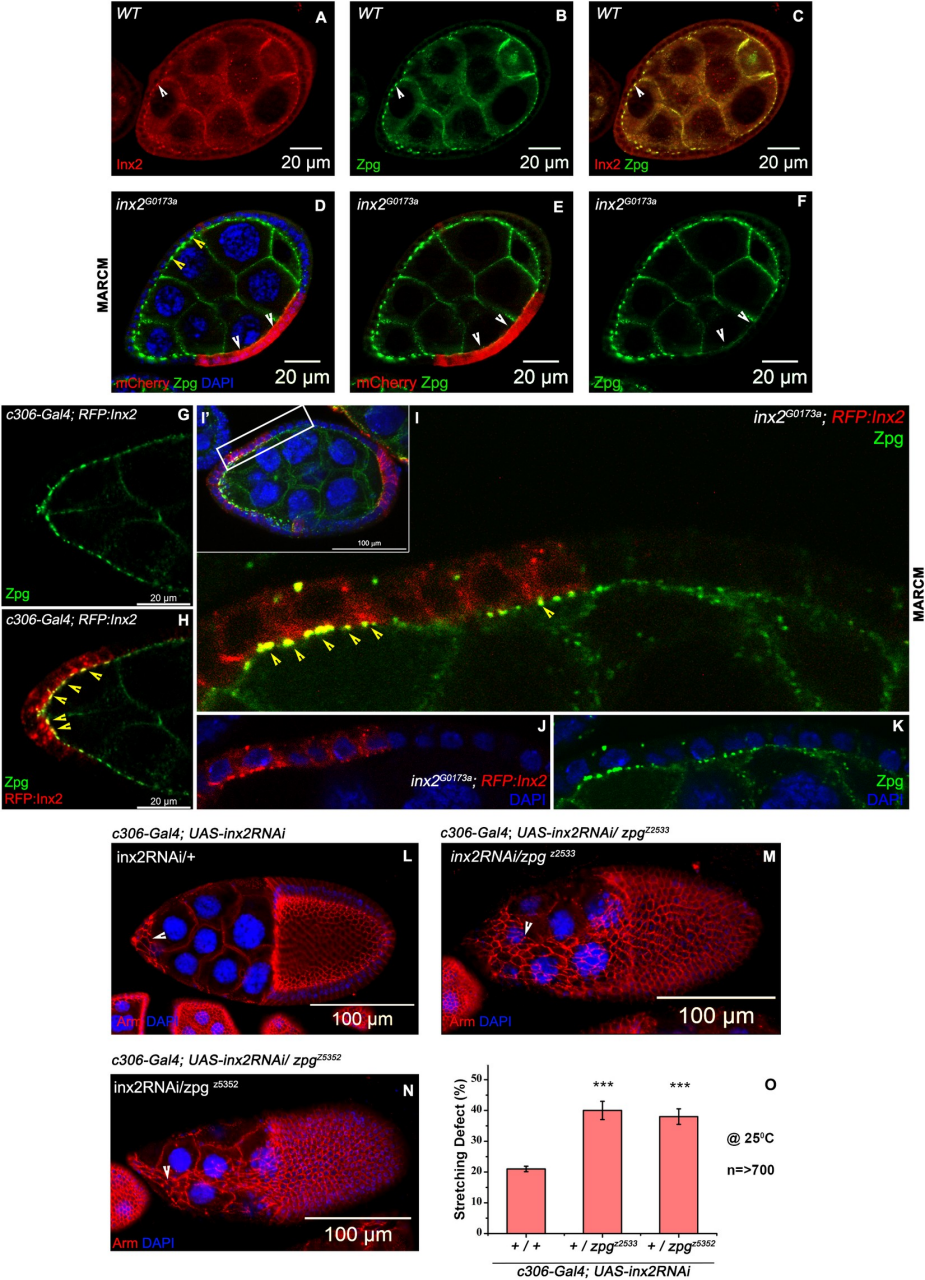
Partial co-localization and genetic interaction between Inx2 and Zpg. **(A-C)** Expression pattern of Inx2 and Zpg in the wild type (CsWT) egg chambers. White arrowhead marks respective proteins. **(A)** Inx2 in Red. **(B)** Zpg in Green. **(C)** Merge of A and B. **(D-F)** MARCM analysis. *inx2*^*G0173a*^ mutant follicle cells are marked with mCherry imaged in Red and Zpg is shown in Green. Note the reduction in the levels of Zpg at the Nurse Cell-Follicle Cell interface (White arrowhead). Yellow arrowhead in D. marks the Zpg expression in the *inx2*^*G0173a*^*/+* follicle cells. **(G-H)** Stage 8 egg chambers of indicated genotype. **(G)** Zpg is in Green **(H)** Merge of Red in RFP:Inx2 and Zpg is in Green. Yellow arrowhead mark the colocalization of RFP:Inx2 and Zpg at the interface of follicle and nurse cell boundary. **(I-K)** MARCM analysis. **(I’)** Stage 8 egg chambers carrying clones of *inx2*^*G0173a*^ mutant AFCs that are overexpressing RFP:Inx2. **(I-J)** Magnified image of the Boxed outline in I’. **(I)** Merge of RFP:Inx2 in Red (J) and Zpg in Green (K). Yellow arrowhead marks the colocalization of RFP:Inx2 and Zpg at the interface of follicle and nurse cell boundary. DAPI is in Blue in J and K. **(L-O)** Inx2 and Zpg genetically interact during the shape transition seen in the AFCs. **(L-N)** Stage 10 egg chambers of indicated genotypes exhibiting stretching defects. Armadillo in Red and DAPI is in Blue. **(O)** Quantification of the stretching defect. Note increase in stretching defect of Inx2-depleted follicle cells in *zpg* heterozygous background. *** represents p-value <0.001. Error bars represent Standard Error of Mean. n indicates the number of egg chambers analyzed.

To test the idea, we co-stained wild type egg chambers with the antibodies against Inx2 and Zpg respectively. Curiously in addition to their subapical localization, Inx2 puncta were also detected at the apical membrane of follicle cells (Fig 7A). While, Zpg localized to the nurse cell membranes and was also detected at the NC-FC junction (Fig 7B) [48]. We also observed a staining overlap between Inx2 and Zpg at the NC-FC junctions suggesting that the two proteins co-localize partially (Fig 7C). Since the overlap is partial, we sought to test if localization of the two proteins is co-dependent. To assess this possibility, we generated homozygous mutant clones of *inx2* and stained the egg chambers with the anti-Zpg antibody. Interestingly, we observed loss of Zpg protein at the NC-FC junction abutting the *inx2* mutant follicle cell (Fig 7D–7F). This observation suggested that partial co-localization of these two proteins could be functionally relevant.

We repeated this experiment with *RFP*:*Inx2* to ascertain if its overexpression in the wild type and *inx2* mutant follicle cells can affect the localization of Zpg. Consistent with our expectation, we observed normal localization of Zpg protein in both the cases with partial co-localization between both the proteins at the follicle cell and nurse cell interface (Fig 7G–7K and S1 Video). These data further support that the structural properties of RFP:Inx2 protein is relatively intact.

To further examine a possible functional connection between Inx2 and Zpg, we decided to evaluate the stretching of Inx2-depleted AFCs (*c306-Gal4; UAS-inx2RNAi)*, in wild type (+/+) and *zpg* heterozygous background (*zpg*^*[z-5352]*^*/+ or zpg*^*[z-2533]*^ */+)*. Consistent with our expectation, simultaneous depletion of Inx2 and Zpg resulted in higher incidence of stretching defects (38±2.5% & 40.3±3.0% of stretching defect compared to 21.3±0.9% observed in wild type background). This experiment was performed at 25°C for moderate expression of RNAi, for details see materials and methods (Fig 7L–7O). Together these data suggest that Inx2 and Zpg interact genetically and might function together to modulate shape change of AFCs.

Based on these observations we wondered if Inx2 and Zpg may work together by forming a heterotypic gap junction channel between the NC-FC cell surfaces. Furthermore, such an interaction could facilitate the flattening of the AFCs. We have previously shown that channel activity of Inx2 mediates calcium flux in the follicle cells to modulate JAK-STAT activity during border cell fate specification. Intriguingly, down-regulation of Inx2 by employing the same Gal4 driver (*c306-Gal4*) not only affected the border cell fate and calcium flux but also the cuboidal to squamous fate transition in the AFCs (Fig 1G–1L). We were therefore curious to check if Inx2 and Zpg function in the somatic cells and nurse cells respectively to modulate the calcium flux in the flattening follicle cells.

### Coordination of calcium flux between the germline and soma

By employing green fluorescent calcium sensor, GCaMP6, we have previously documented that communication between follicle cells is mediated by calcium [24]. Our data showed initiation of a periodic wave of free calcium in a random subset of outer follicle cells, which spread to neighboring follicle cells over time [24]. We also showed that the intensity and transmission of the calcium waves were sensitive to levels/activity of Inx2. However, these studies did not address if the calcium flux is also present in the germline cells and if so, whether the follicle cells and germ-line cells communicate via calcium flux. In the following we have first examined if nurse cells display calcium flux and how nurse cell specific activity of Zpg can influence the calcium flux in the nurse cells and the overlying follicle cells.

**Fig 8:**
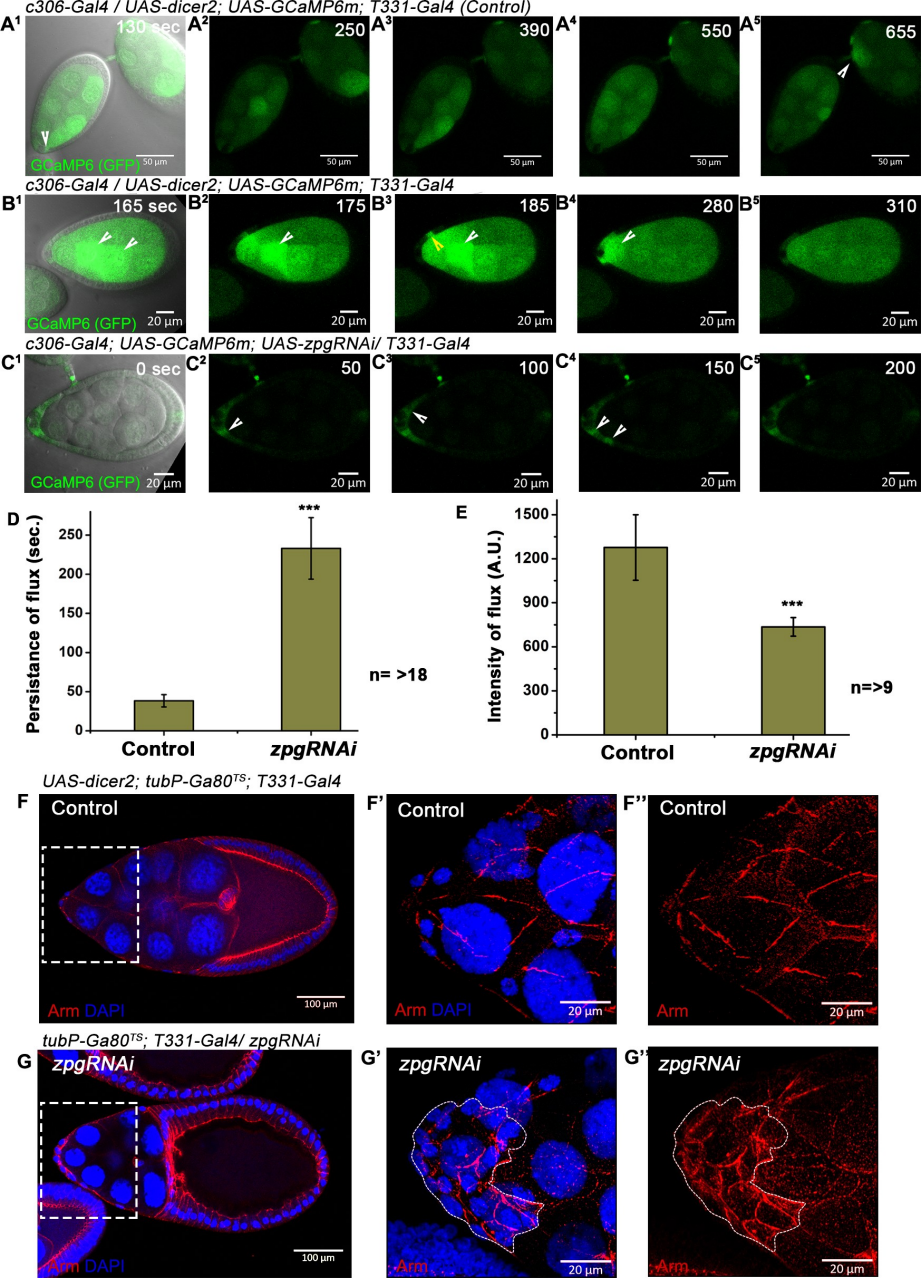
Germline-soma interaction via calcium flux is mediated by the gap junction proteins, Inx2 and Zpg. **(A-C)** Time lapse imaging of the egg chambers depicting calcium flux in the indicated genotypes. **(A**^**1**^**-A**^**5**^**)** Please note the sporadic instances of calcium flux in the nurse cells. Arrowhead in A^1^ indicates the flux in the nurse cells and adjacent follicle cell are coincident. Please note the follicle cell flux in A^4^ precedes gradual flux build up in the neighboring Nurse cell (Arrowhead in A^5^). Note the flux signal in the Nurse cells is gradually extending from the side facing the follicle cells towards its center. **(B**^**1**^**-B**^**5**^**)** Indicates instances of flux moving between the nurse cells (White arrowhead). Yellow arrowhead marks the flux in the follicle cells that follows the flux in neighboring nurse cells. Please note the flux in the follicle cells in B4 is coincident with high flux in the neighboring nurse cell. **(C**^**1**^**-C**^**5**^**)** Down regulation of Zpg function in the nurse cells completely blocks the germ line flux. Note the residual flux in the follicle cells indicated by white arrowhead. **(D-E)** Quantitation of calcium flux in the follicle cells with respect to persistence in D and intensity in E. Control and *zpgRNAi* is the genotype represented in panel B and C respectively. n represents the number of egg chambers analyzed. *** represent p-value <0.001. Error bars represent Standard Error of Mean. **(F-G)** Downregulation of Zpg function in the nurse cells non-cell-autonomously affects the stretching of follicle cells. Egg chambers of indicated genotypes. Region under white dotted box in F and G are shown in F’-F” and G’-G” respectively. Armadillo is in Red and DAPI is in Blue. Please note that follicle cells under the white dotted outline in G’ and G” marks the unstretched follicle cells in stage 10 egg chambers.

To probe these possibilities systematically we expressed genetically encoded GCaMP6 reporter both in the wild type nurse cells (*T331-Gal4*) and AFCs (*c306-Gal4*). Expression of GCaMP6 reporter using *T331-Gal4* exhibited readily detectable level of fluorescence in the nurse cells (Figs 8A and 8B and S2 Video and S3 Video). As the oogenesis progressed, we observed stochastic increase in the flux intensity in a few nurse cells over the basal level suggesting a presence of nurse cell specific calcium flux.

Importantly, in several instances, a conspicuous increase of flux intensity in a given nurse cell also strongly correlated with a higher level of fluorescence in the neighboring follicle cells (Fig 8A^4^–8A^5^). In addition, we also observed movement of calcium flux between the nurse cells (Fig 8B^1^–8B^5^). This observation suggested that calcium flux that originates in the nurse cells might induce a similar flux in the neighboring follicle cells (Fig 8B^2^–8B^4^). To rule out the possibility of direct physical transfer of the reporter molecule, we overexpressed GCaMP6 reporter only in the nurse cells. In the absence of GCaMP6 in the FCs, calcium flux was never detected in the overlying follicle cells arguing against a direct physical movement of the GCaMP6 reporter across the germline-soma boundary (S4 Video).

Altogether these data suggested that NCs actively communicate with the FCs at the later stages of oogenesis. We have previously shown that depletion of Inx2 function in the follicle cells inhibits the calcium flux in the follicle cells [24].

Next, we examined if the germline specific calcium flux is sensitive to presence of Zpg. Overexpression of *zpgRNAi* in the germline nurse cells and anterior follicle cells (*c306-Gal4; T331-Gal4*) completely obliterated calcium flux in all the nurse cells including the basal germline fluorescence but exhibited flux in the follicle cells (Fig 8C^1^–8C^5^ and S5 Video). This data suggested that Zpg plays a significant role in the initiation and modulation of calcium flux in the germline nurse cells.

### Zpg regulates the calcium flux and the cell shape change in the overlying follicle cells

**Fig 9:**
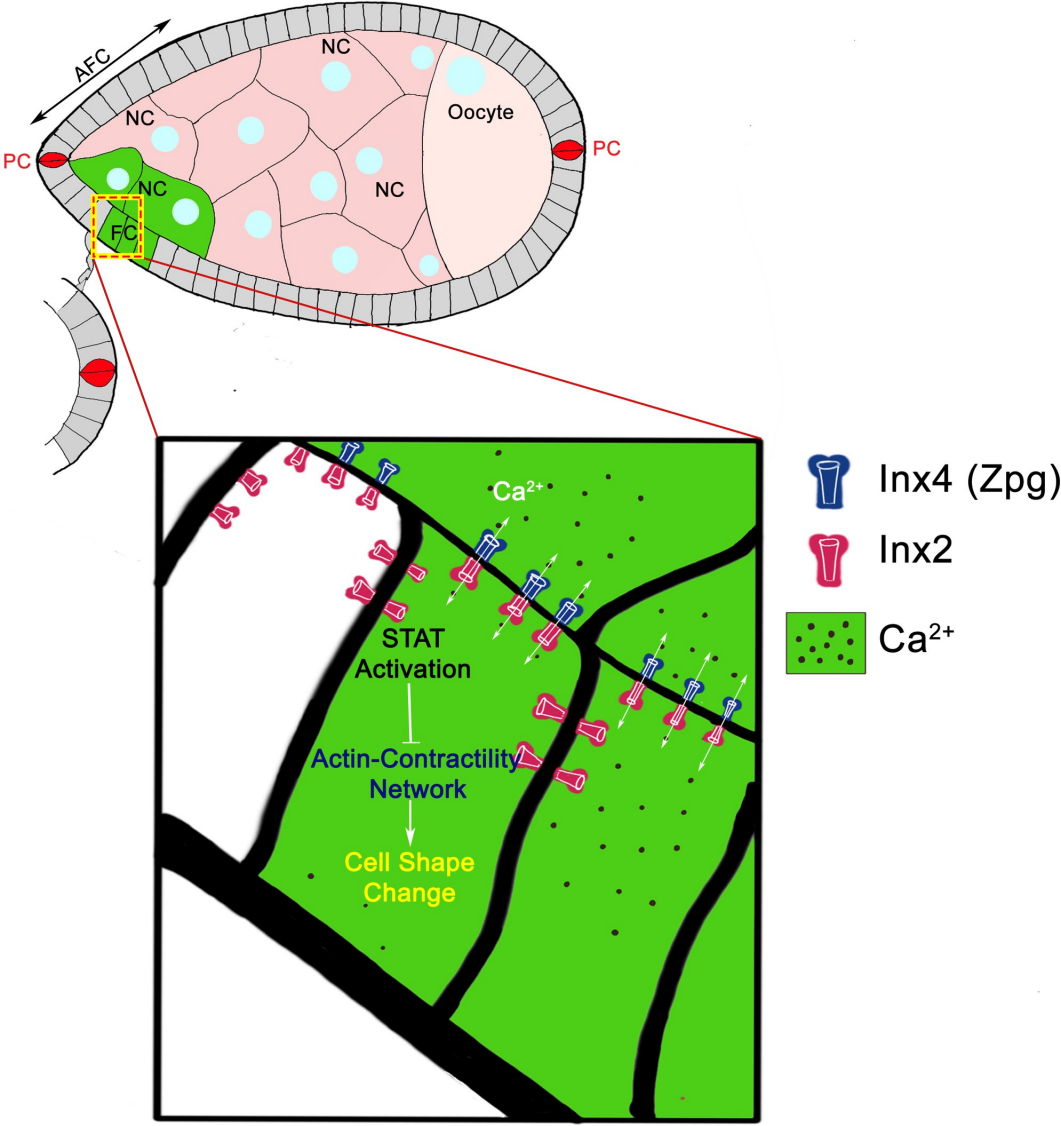
Schematic of the proposed model. Stage 8 egg chambers where abbreviation NC stands for Nurse cells, FC for Follicle cells and PC for Polar cells. The NC and FC shaded in Green indicate the calcium flux. The boxed region of FC in the upper panel is magnified below. Heteromeric GJ channel at the germline (Zpg)/ soma (Inx2) interface stimulate calcium flux in the anterior follicle cells thus activating STAT. Activated STAT negatively regulates the actin contractibility in FC to potentiate cell shape change transition from Cuboidal to Squamous fate.

Next, we examined how the flux dynamics in the follicle cell changes when Zpg function is compromised in the nurse cells. Reduction in the Zpg activity did not influence the basal level of calcium flux in the follicle cells enveloping the Zpg-depleted nurse cells. However, when Zpg was down regulated in the nurse cells, the duration of the flux in the follicle cells was significantly longer (46.63±7.87 sec) compared to the control (7.66±1.58 sec) (Fig 8D). While the intensity of the flux in the overlying follicle cells was significantly reduced (821.24±98.0 a.u.) unlike that observed in the control follicle cells adjacent to wild type nurse cells (1415.91±126.6 a.u.) (Fig 8E). So, compromising *zpg* function in the nurse cells results in dampened yet longer lasting calcium flux in the overlying follicle cells. As Zpg seems to affect the dynamics of calcium flux in the follicle cells, we were curious to know if it also influences the follicle cell flattening. Over expression of *zpg RNAi* using late driver (*T331-Gal4; tub-Gal80*^*ts*^) resulted in follicle cells that were unable to stretch as opposed to the control egg chambers (*T331-Gal4*; *tub-Gal80*^*ts*^*; UAS-dicer2)* (Fig 8F and 8G). In these egg chambers, we also observed a wavy oocyte cortex that has been recently reported suggesting that our *zpg RNAi* construct was indeed working on the expected lines [34]. Importantly, the total number of border cells in migrating cluster was not significantly altered when Zpg was down regulated in the nurse cells (S7A–S7C Fig), nor was the follicle cell morphogenesis affected when Zpg function was down regulated in the follicle cells (S7D–S7G Fig). Altogether these data suggest that Zpg and Inx2 function in the germline and soma respectively to mediate the cuboidal to squamous cell shape change of AFCs during *Drosophila* oogenesis (Fig 9).

## Discussion

Morphogenesis involves complex cellular interactions, regulated via short- or long-range influences, which operate both at the local as well as global level. Interactions between different cell types have acquired extra-ordinary significance during proper execution of complex morphogenetic processes including organogenesis. Interacting cellular partners of heterogenous origins participate in generation of a number of coordinated cues. Such cues either biochemical or mechanical in nature, operate both in localized and widespread manner to ultimately orchestrate the formation of simple as well as complex multicellular assemblies. Variety of signals that control the process, and underlying mechanisms that synthesize as well as propagate such signals are being investigated in different developmental contexts.

Here we demonstrate that Inx2, a gap junction protein, controls cuboidal to squamous cell shape transition in the follicular epithelium. Effect of Inx2 is mediated by its ability to regulate STAT activity, which in turn can influence the cytoskeleton. Supporting the conclusion, changes in STAT or alterations in the cytoskeletal machinery components, can either enhance or mitigate the phenotypic consequences induced by the loss of Inx2. Importantly, Inx2 engineers this shape transition likely by acting as a component of a calcium channel. It seems to achieve this by partnering with Zpg, another gap junction protein, belonging to the same family.

Gap junction proteins have been shown to be required in a number of different biological processes including establishment of epithelial integrity and morphogenesis. For example, Inx2 has been shown to control formation of proventriculus, during foregut formation in the flies [49]. Previous reports have also suggested that Inx2 can form homomeric as well as heteromeric gap junction channels [32,49,50]. However, the molecular mechanism of Inx2 mediated epithelial morphogenesis has remained elusive.

This study advances our mechanistic understanding of how the gap junction channels contribute to morphological changes in the epithelial cells. Though calcium has been implicated in regulation of cell shape transition in various developmental contexts, the precise molecular mechanism underlying calcium dependent cell shape transition has not been documented. Our findings demonstrate that calcium dependent JAK-STAT signaling regulates Myosin distribution to engineer cell shape transition. Interestingly we have previously shown that the same molecular cassette is used for proper specification of BCs. Together these data suggest that Inx2-zpg mediates the calcium flux in the follicle cells, which probably regulates JAK-STAT signaling in a context specific manner to achieve diverse outcomes.

### Communication between the germline and soma assists epithelial morphogenesis

A heterotypic combination of Inx2-Zpg gap junction channel has been reported to regulate germline-soma interaction during early *Drosophila* spermatogenesis [51]. In addition, heterotypic combination between Inx2 with other somatic gap junction protein, Inx3 has been reported in developing *Drosophila* embryo during the regulation of epithelial morphogenesis [52]. Here we have assayed the ability of a putative Inx2-Zpg heterotypic GJ channel responsible for cell shape transition underlying egg chamber morphogenesis. Our results suggest that via Zpg, nurse cells induce the follicle cells to elevate free calcium levels as germline specific depletion of Zpg affects the absolute intensity and duration of calcium flux in the follicle cells. We thus believe that optimum amount of calcium is critical for mediating proper shape transition of AFCs from cuboidal to squamous fate. Although, the nurse cells exhibit high level of GCaMP6 fluorescence, signal transmission to the adjacent follicle cells is relatively infrequent. This observation suggests that the transmission of the signal via Inx2-Zpg is spatially and temporally calibrated during oogenesis. Nurse cells are relatively of a larger size and, as such a direct physical contact may be established between a single nurse cell with a number of follicle cells via formation of a heterotypic GJ channel(s). Contact dependent assembly of Inx2-Zpg channel likely confers selectivity on the release and strength of the calcium flux. Taken together our data are consistent with the calcium flux being a crucial player during this process while the precise identity of the signal, remains to be determined. Whether calcium itself is the elusive ‘signal’ needs to be investigated further. It is possible that the ‘signal’ is more complex and consists of several components with calcium being one of the pieces of the puzzle. In such a scenario, future experiments ought to focus on uncovering the identity of the other components of the signal. It will also be important to determine as to how the non-autonomous ‘signal’ secreted by the nurse cells, is conveyed to the somatic neighbors to modulate the calcium flux. Follicle cells express many gap junction proteins including Inx2 on their cell surface and are competent to communicate with one another. Thus, possible contribution of neighboring follicle cells, not in direct contact with the nurse cells, may also be an important determinant.

### Endocytosis of membrane component might be crucial for stretching process

Recently product of *tao* gene has been shown to regulate follicle cell flattening by modulating the removal of lateral membrane component Fas2 by endocytosis [53]. On similar lines, our data shows that Inx2 permits the disassembly, and perhaps also the disappearance of adherens junction component DE-cadherin from cuboidal cells to facilitate the follicle cell stretching. Since calcium is known to regulate the rate of endocytosis, we propose that calcium may regulate the levels of DE-Cadherin via endocytosis to mediate the shape change in the AFCs. Partial rescue of *inx2* phenotype in the heterozygous *shg* mutant background suggests that the accumulation of DE-Cadherin is indeed functionally connected to the phenotype. Further experiments will help reveal mechanistic connection between calcium flux and adherens junction assembly and its influence on cell shape transitions.

### Relevance for skin development: A perspective

Epidermal stratification during development of mammalian skin depends on cell division, collective migration and controlled differentiation. Together these three processes are responsible for the basal to squamous fate transformation [54]. It is reported that this fate change is also accompanied by increase in the levels of GJ proteins and inter- as well as intracellular calcium [55,56]. Our results have implicated combined involvement of GJ proteins and calcium flux in mediating cell shape changes. It will be worthwhile to examine if similar mechanisms are employed by the differentiating basal cells. Intriguingly, several skin disorders have been linked to mutations in connexins (vertebrate counterparts of GJ proteins). Our results thus provide a readily accessible genetic platform to compare and contrast the molecular mechanisms underlying epithelial cell shape transitions in diverse cellular contexts.

## Materials and methods

### *Drosophila* stocks and genetics

Fly stocks and crosses were maintained under standard conditions (25°C) unless otherwise stated. *c306-Gal4 (BDSC-3743)*, *hsFLP; P[Actin-<17b >Gal4]*, *T331-Gal4*, *c289b11-Gal4* (BDSC-6981), *GR1-Gal4* (BDSC-36287) used for the expression of various transgenes were crossed with UAS-mCD8:GFP reporter to test if the drivers were functional. *T331-Gal4* were a gift from Prof. Daniel St. Johnston (The Gurdon Institute, Cambridge University). *UASp-Moesin*:*Cherry*, *P{UAS-Inx2}*, *P{UAS-RFP*:*Inx2}* were kindly provided by Prof. Andrea Brand (The Gurdon Institute, Cambridge University). *P{UAS-Inx2}* was also provided by Dr. Reinhard Bauer (University of Bonn, Germany). *P{UAS-Itpr}* and *P{UAS-Orai}* were received from Prof. Gaiti Hasan (NCBS, India). *P{w[+mC] = lacW}BB127* was kindly provided by Prof. Trudi Schupbach. UAS-tdTomato was provided by Prof. Krishanu Ray (TIFR, India). *FRT42B shg*^*6115*^*/CyO* and *FRT42D shg* ^*[p34-1]/*^*SM6B* a gift from Dr. Richa Rikhy (IISER Pune) [57]. UAS Asrij on 3^rd^ chromosome was a gift from Prof. Maneesha Inamdar (JNCASR, Bangalore, India). *inx2* alleles *P{w[+mC] = lacW}inx2*^*G0173a*^(DGRC-111858), P{w[+mC] = lacW}*inx2*^*G0059*^ (DGRC-114609) were obtained from Drosophila Genetic Resource Centre, Kyoto. *UAS-inx2RNAi* (BDSC-29306) and *P{EPgy2}Stat92E*^*EY14209*^*/TM3 Sb Ser* (BDSC-20915), P{w[+mC] = lacW}*Dad*^*[j1E4]/*^TM3, Sb[1] (BDSC-10305), *y*^*1*^ *hop*^*27*^*/FM7c* (BDSC-8493), *cn*^*1*^ *bw*^*1*^ *sp*^*1*^ *zip*^*1*^*/CyO* (BDSC-4199), *ry506 P{PZ}Stat92*^*E06346*^*/*TM3, ryRK Sb1 Ser1 (also referred as *Stat92E*^*P1681*^) (BDSC-11681), *sqhRNAi*, *zpg*^*[z-2533]*^ *st[1]/TM3, Sb[1]* (BDSC-9918), *bw[1]; zpg*^*[z-5352]/*^*TM6B, Tb[1]* (BDSC-9919), *statRNAi* (BDSC-33637), *w[1118]; P{w[+mC] = UAS-MLCK*.*ct}87*.*11/CyO*, P{ry[+t*] = elav-lacZ.*H}YH2* (BDSC-37528), P{w[+m*] = NRE-EGFP.*S}5A* (BDSC-30727), *UASp-myrGFP* (BDSC-58721), *UAS-GMA* (BDSC-31775) lines were obtained from Bloomington Drosophila Stock Centre, Indiana University.

S1 Table has the list of genotypes organized figure wise.

### Immunohistochemistry

Ovary dissection, fixation and staining were performed using standard protocol [58]. The following primary antibodies were used: mouse anti-Armadillo [N27A1] [Developmental Studies Hybridoma Bank (DSHB), 1:50; rat anti-DE-Cadherin (DCAD2) 1:50 (DSHB); rabbit anti-Zpg 1:2000 and guinea pig anti-Inx2 1:1000 (gift from Guy Tanentzapf), rabbit anti-phospho-Myosin Light Chain 2 (Ser19) Antibody 1:10 (catalog No: 3671, Cell signaling), and rabbit anti- Zipper 1:200 (gift from Eric Wieschaus), mouse anti-β-Gal (40-1a, DSHB) 1:100, Hindsight (1G9, DSHB) 1:5. Secondary antibodies conjugated with Alexa-488 and Alexa-568 (Molecular Probes) were used at 1:250 and 1:400 dilutions respectively. Rhodamine Phalloidin staining was performed using standard procedure [58].

### Statistics

Two-tailed T-Test of unequal variance was used in Excel to assess statistical significance. Graphs were plotted using either Origin Pro 8, Microsoft Excel or GraphPad Prism. * Indicates p value <0.05, ** indicates <0.01, *** indicates <0.001. Standard Error of Mean (S.E.M.) was calculated and represented for each data.

### Fly genetics and Mutant analysis

For mutant analysis, *P{w[+mC] = lacW}inx2*^*G0173a*^ *FRT 19A/FM7* flies were crossed with *hsFLP*,*tubP-GAL80*,*neoFRT19A*; *actin-Gal4 UASp-Moesin*:*Cherry/TM3Sbe*. F1 flies were subjected to heat-shock for 1 hour thrice a day for 3 consecutive days. Flies were incubated at 25°C for 5 days followed by fattening at 25°C. For MARCM rescue experiment *UAS-Inx2* was combined with *P{w[+mC] = lacW}inx2*^*G0173a*^ *FRT19A/FM7* stock and crossed as mentioned earlier.

For flip-out clonal analysis, flies of genotype *hsFLP UAS-mCD8*: *GFP; actin<flipout>GAL4* was crossed with *UAS-inx2RNAi*, *UAS-RFP:Inx2*, *UAS-MLCK*.*CA*, *UAS-sqhRNAi*. F1 flies of appropriate genotype were subjected to heat-shock for 1 hour duration thrice a day for 3 consecutive days. The flies were dissected on the 6^th^ day after the last heat shock and ovaries were immunostained with Inx2 or Zipper antibody.

For experiments in which the F1 genotype was lethal (*zpgRNAi* with *T331-*Gal4), temperature-sensitive Gal80 was used to downregulate Gal4 activity during early development. Crosses were kept at 16°C till progenies emerged. F1 flies were shifted to 31°C (restrictive temperature) for 18 hours to inactivate GAL80 before dissection.

In all of the RNAi experiments, desired F1 genotype flies were fattened at 29°C for 18 hrs. *UAS-Moesin*:*GFP or UAS-mCD8*:*GFP* were used to normalize the number of UAS constructs in the cross. All imaging were performed with CZI Apotome & LSM 710 confocal microscope.

### Quantification of Stretching defect

To quantify the stretching defect, only stage 10 egg chambers, where all anterior cells are supposed to exhibit complete stretching were considered. Egg chambers in which anterior end of egg chambers retain cuboidal cells or smaller cells which lose their cuboidal shape but failed to enlarge as stretched cells were considered as defective in stretching. The percentage stretching defect represents the number of the stage 10 chambers exhibiting stretching defect of AFCs compared to the total number of stage 10 egg chambers analyzed for that particular genotype.

### Quantification of Neolamination

To quantify the Neolamination defect, late stage 10- early stage 11 egg chambers were considered. At these stages, the apical side of the border cell clusters physically attaches with the migrating centripetal cells. Egg chambers where the border cell clusters failed to attach to the centripetal cells were referred to as defective in neolamination. We only considered those egg chambers where the border cell clusters were in the vicinity of the oocyte.

### 10XSTAT-GFP/ NRE-GFP intensity calculation

For the quantification of 10XSTAT-GFP/ NRE-GFP in the follicle cells anterior end of stage 8 egg chambers were captured keeping polar cells at the center plane. Excluding the first 3 cells from the polar cells, intensity calculation was restricted to the next group of 3 cells on either flank outlined on the basis of Armadillo staining/ Phalloidin and the mean intensity of 10XSTAT-GFP/ NRE-GFP was extracted.

### Cell area/size measurement

For measuring cell size (area), clones (GFP+) and non-clone cells (GFP-) were compared keeping equal number of cells per group in each egg chamber. Cell number was counted with help of DAPI staining and cell membrane was outlined by marking Arm staining. Further analysis was performed in Image J software where cell area was extracted out from the outlined total area divided by number of cells to finally calculate area/cell (cell size). At least 10 cells were considered for each group (GFP+/GFP-) per egg chamber.

### Calcium imaging

*P{UAS-GCaMP6m*} (BDSC-42748) line was over expressed by *T331-Gal4* & *c306-Gal4* for detecting free Ca^2+^ in the nurse cells & follicle cells. 12 bit images of frame size 512X512 were captured with CLSM 710 (Zeiss) with Plan-Apochromat 63X oil immersion objectives, N.A 1.4. Argon laser line 488 nm was used for GCaMP6 reporter. Fast continuous live imaging with 6.3μm pixel dwell at frame interval 5 seconds for 5–6 minutes was used for recording the calcium flux. Sequential imaging was performed using Argon 488 laser with time interval 7 second per frame for recording calcium flux. *c306-Gal4/UAS-dicer2; UAS-GCaMP; T331-Gal4* was used to visualize the activity of calcium simultaneous in the germline Nurse cells and Anterior follicle cells. The experimental genotypes expressed *UAS-zpgRNAi* instead of *UAS-dicer2* in the control. The flies were fattened at 29C for 19.5 hours. Live imaging was carried out as reported previously [24].

12 bit images of frame size 512X512 were captured with CLSM 710 (Zeiss) with 40X oil immersion objectives. Argon laser (488 nm) was used for GCaMP6m reporter (pinhole diameter 3.5 AU). Time lapse imaging was carried out at intervals of 5 secs for at least 10 minutes. For flux intensity quantification, we used Image J. The individual follicle cells were marked on the basis of DIC image. The mean basal intensity and the maximum flux intensity was quantified for each cell showing flux. The maximum flux intensity was subtracted from the basal intensity for each cell, and was compared across the two genotypes. The number of timepoints for which the flux could be seen was used to plot the persistence graph.

## Supporting information

S1 Fig Inx2 mediates the stretching of the anterior follicle cells during cuboidal to squamous shape transition. **(A-C)** Egg chambers of indicated stages depicting the expression pattern of *c306-Gal4*. GFP is in Green, Inx2 in Magenta and DAPI in Blue in A. Please note expression of *c306-Gal4* in the anterior follicle cells. **(D-L)** Clonal over expression of *inx2RNAi* mediated by *actin[Flipout] -Gal4*. Clones are marked in Green. **(D-F)** *inx2RNAi* overexpressing clone outlined with a dotted line. Inx2 is in Magenta. DAPI is Blue in D. Please note reduction in the Inx2 level in the RNAi expressing clones in F. Yellow arrow head mark the residual Inx2 protein in the clone. **(G-L)**. Armadillo is in Red and DAPI is in Blue. Arrows point to unstretched cells in H. **(J)** Small sets of clones at the anterior end (Arrows) exhibiting stretching defect. **(J-L)** Please note moderate phenotype where follicle cells (arrows) have lost their cuboidal shape but are unable to stretch.
(TIF)

S2 Fig Inx2 function in the anterior follicle cells mediates the cuboidal to squamous shape transition. **(A-M)** MARCM analysis with *inx2* mutants. (**A-E)** mCherry in Red marks the *inx2*^*G0059*^ mutant clones. Arm is Green in A-C and DAPI is Blue in A and D. Arrowhead marks the unstretched follicle cells. Arrow points to the posterior clones, which appear to be normal in A & B. **(D-G)** *inx2* mutant alleles are near nulls (protein). Please note strong down regulation of Inx2 protein in *inx2* mutant posterior clones *inx2*^*G0059*^ (D-E) and *inx2*^*G073a*^ (F-G). The clones are highlighted in dotted outline, marked by presence of mcherry in Red and Inx2 is in Green. **(H-J)** Stage 10 egg chambers with *inx2*^*G073a*^ germline clones of Inx2 marked in Red, DAPI in Blue and Arm in Green. Please note the egg chambers have normal morphology. **(K-M)** Surface view of *inx2*^*G073a*^ AFCs rescued by overexpression of *Inx2 cDNA*. mcherry is in Magenta marking the *inx2*^*G073a*^ AFCs. Arm in Green and DAPI in Blue. **(N-R)** Egg chambers of indicated genotypes. **(N-O)** Armadillo in Magenta in N & O, GFP in green denotes the expression domain of *c289b11-Gal4* in N and *cy2-Gal4* in P & Q. and DAPI in blue. **(O & R)** Please note that depletion of Inx2 function in the main body follicle cells in O or in centripetal cells in R doesn’t impede shape change of anterior follicle cells. Phalloidin is Red, in P, Q and R. Please note that depletion of Inx2 function in the main body follicle cells doesn’t impede shape change of anterior follicle cells.
(TIFF)

S3 Fig Inx2 loss does not seem to influence the fate of the AFCs. **(A-C)** Stage 10 egg chambers of indicated genotypes. *dad-lacz* and DAPI imaged in Green and Blue respectively. Note that *dad-lacz* marks the squamous and centripetal follicle cells. Arrowhead mark the squamous fate **(B-C)** MARCM analysis. Stage 10 egg chambers harboring *inx2*^*G0173a*^ mutant follicle cells marked in Red by mcherry and highlighted by dotted lines. The unstretched anterior follicle cells express *dad-lacz*. **(D-E**) Egg chambers of Indicated genotypes. *BB127-lacz* in Magenta and Arm in Green. White dotted outline marks the unstretched AFCs in E and F. Arrow in D & F marks the *BB127-lacz* expression in the centripetal cells. Please note absence of *BB127-lacz* expression in the unstretched AFCs (dotted outline). **(G-I)** Transcription factor Eya is excluded from the main body follicle cells. Over expression of *inx2RNAi by c306-Gal4* exhibit defect in shape transition in AFCs (boxed in H & I). Red is Eya, Green is Phalloidin and DAPI in blue. Please note Eya is expressed in the AFCs that exhibit stretching defect. **(J-Q)** Inx2 functions independent of Notch to mediate the stretching of the anterior follicle cells. **(J-L)** Clonal over expression of *inx2RNAi* mediated by *actin [Flipout] Gal4* in stage 8 egg chamber. Clonal area is outlined and marked in Green. Hindsight is in Red and DAPI is in Blue. Please note that there is no difference in the level of Hindsight protein between the clones and non-clone follicle cells. **(M-P)** Stage 8 egg chambers with white dotted line outlining the AFCs that would stretch to acquire squamous fate. Control is *c306-Gal4*/ *UAS-dicer2; NRE-*GFP and *inx2RNAi* (*c306-Gal4*; *NRE-*GFP/ UAS-*inx2RNAi*). *NRE*-GFP expression is in Green, Arm in Red and DAPI in blue. **(Q)** Quantification of the *NRE*-GFP of control and *inx2RNAi*. ‘n’ stands for the number of egg chambers analyzed. Error bars indicate standard deviation. p is the level of significance.
(TIFF)

S4 Fig RFP:Inx2 doesn’t impede neolamination. **(A-C)** images of Border cell cluster near the oocyte boundary. A’. B’ and C’ are the magnified image of A, B and C respectively. Arm is in Black. Blue Arrows in A’ and B’ indicate the neolaminating border cell clusters. Double end yellow arrow in C’ marks the border cells that exhibit neolamination defect. **(D)** Quantification of Neolamination Defect in the indicated genotypes. Please note that unlike *inx2RNAi*, overexpression of RFP:Inx2 in border cells clusters exhibited normal neolamination. ‘n’ stands for the number of egg chambers analyzed. p is the level of significance. ns stands for not significant.
(TIFF)

S5 Fig STAT affects the cuboidal to squamous shape change of AFCs. **MARCM analysis**. **(A-C)** *stat92E*^*P1681*^ mutant clones are marked in Green, Armadillo in Red and DAPI in blue. Arrow marks the unstretched follicle cells in the mutant clones. **(D-F)** Overexpression of STAT fails to rescue the Inx2RNAi induced phenotype in AFCs. Quantification of stretching defects in the stage 10 egg chambers of the indicated genotypes. “n” is the number of egg chambers evaluated. Please note that there is no significant difference (ns) in the percentage of observed phenotype for the two genotypes in D and F. **(G-K)** Overexpression of Asrij activates JAK-STAT signaling in the follicle cells. **(G-J)** Stage 8 egg chambers of the indicated genotypes. *10XSTAT*-GFP expression is in Green in H & J DAPI is Blue. in G and I. **(K)** Quantification of the *10XSTAT*-*GFP*. Please note increase in the levels of *10XSTAT*-*GFP* in the follicle cells overexpressing Asrij. ‘n’ indicates the number of egg chambers analyzed. *** represent p-value <0.001. Error bars represent Standard Error of Mean.
(TIFF)

S6 Fig STAT modulates the levels of Zipper and Phospho-myosin to facilitate shape transition in AFCs. **(A-B)** AFCs of the Wild type egg chambers. (A) DAPI in Blue. (B) Zipper expression is in Magenta. Please note that Zipper levels are lower in the follicle cells. High level of Zipper is detected in the stalk cells. **(C-G)** Mosaic analysis-employing *actin [Flipout] Gal4* resulting in overexpression of *statRNAi* in clones. The clones are marked in Green. **(C-E)** Representative image of the whole egg chamber exhibiting *statRNAi* induced stretching defect Zipper is in Magenta and DAPI in Blue. Inset in C is the representative image of the corresponding egg chamber. Please note Zipper enrichment in cells exhibiting stretching defect in D & E (marked by yellow arrow head). The border cell cluster in the center exhibits high level of Zipper staining in C to E. **(F-H)** Rescue of stretching defect of STAT depleted follicle cells when Zip levels (*zip*^*1*^*/+*) are reduced. *statRNAi* overexpressing clones outlined in Green. Please note the rescue in cell size of STAT depleted AFCs in G. **(H)** Quantification of Stretching defect for the indicated genotypes. **** represents p-value <0.0001. n stands for the number of egg chamber analyzed. ‘ns’ stands for not significant. **(I-J)** Phospho-myosin in the wildtype follicle cells. Please note transient accumulation of Phospho-myosin in the cells undergoing shape change. **(K-N)** Representative image of the whole egg chamber exhibiting *statRNAi* induced stretching defect. **(K-N)** pMyosin in Magenta. M and N are magnified inset of the rectangular outline in L. White Arrowheads in N marks retention of pMyosin in follicle cells exhibiting stretching defect.
(TIFF)

S7 Fig Down regulation of Zpg function in the nurse cells doesn’t affect the border cell fate. **(A-B)** Egg chambers of indicated genotypes. Armadillo is in Red and DAPI in Blue. **(C)** Quantification of number of border cells in migrating clusters of control (A) and *zpgRNAi* (B). ‘ns’ indicates statistically insignificant. ‘n’ stands for number of clusters analyzed. **(D-H)** Down regulation of Zpg function in the follicle cells doesn’t affect shape transition of cuboidal cells to squamous fate. Stage 10 egg chambers of indicated genotypes. GFP is in Green in D, F and G. DAPI is in Grey and Arm in Magenta.
(TIFF)

S1 Video Colocalization of Zpg and RFP:Inx2 in a subset of anterior follicle cells. 3D projection of stage 8 egg chamber of genotype *c306-Gal4*: *UAS-RFP*:*Inx2* (Red) stained for Zpg (Green). Please note the colocalization of Zpg and RFP:Inx2 in Yellow at the anterior side of the egg chambers. Scale bar is on top left.
(MOV)

S2 Video Germline nurse cells and follicle cells exhibit sporadic calcium fluxes. Time lapse imaging of an egg chamber (Nomarski) over expressing genetically encoded calcium indicator (Green) both in the nurse cells and the anterior follicle cells. Genotype: *c306-Gal4/ UAS-dicer2; UAS-GCaMP6; T331-Gal4*. Please note instances where flux in the nurse cell is coincident with the flux in adjacent follicle cells. There are instances where the flux in follicle cells potentiates building up of flux in the neighboring nurse cells. Time stamp is in 00 minutes: 00 seconds.
(MOV)

S3 Video Transfer of calcium fluxes between the Germline nurse cells. Please note instances where flux (Green) in the nurse cell is coincident with the flux in adjacent follicle cells. Time lapse imaging of an egg chamber (Nomarski) expressing genetically encoded calcium indicator (Green). Genotype: *c306- Gal4/ UAS-dicer2; UAS-GCaMP6; T331-Gal4*. Time stamp is in 00 minutes: 00 seconds.
(MOV)

S4 Video GCAMP6 molecule doesn’t physically transfer between the nurse cell and the follicle cells. Time lapse imaging of an egg chamber (Nomarski) overexpressing genetically encoded calcium indicator (Green) only in the nurse cells. Genotype: *UAS-GCaMP6; T331-Gal4*. Please note that the calcium fluxes between the Germline nurse cells don’t activate any flux in the neighboring follicle cells. Time stamp is in 00 minutes: 00 seconds.
(MOV)

S5 Video Zpg mediate flux transfer between the nurse cells. Time lapse imaging of an egg chamber (Nomarski) overexpressing genetically encoded calcium indicator (Green) and *zpgRNAi* in the nurse cells and anterior follicle cells. Genotype: *c306-Gal4; UAS-GCaMP6; UAS-zpgRNAi/ T331-Gal4*. Please note the complete absence of calcium fluxes in the Zpg-depleted Germline nurse cells. Time stamp is in 00 minutes: 00 seconds.
(MOV)

S1 Table List of fly genotypes used in the experiments organised figure wise. (PDF)

## References

[1] Morphogens Tabata T., their identification and regulation. Development. 2004;131: 703–712. doi: 10.1242/dev.01043 14757636

[2] Schöck F, Perrimon N. Molecular Mechanisms of Epithelial Morphogenesis. Annual Review of Cell and Developmental Biology. 2002;18: 463–493. doi: 10.1146/annurev.cellbio.18.022602.131838 12142280

[3] Freshney RI. Introduction. Culture of Epithelial Cells. New York, USA: John Wiley & Sons, Inc.; 2003. pp. 1–30. doi: 10.1002/0471221201.ch1

[4] Reuter R. Dellmann’s Textbook of Veterinary Histology. 6th edition—Edited by Eurell J, Frappier BL. Australian Veterinary Journal. 2007;85: 310–310. doi: 10.1111/j.1751-0813.2007.00177.x

[5] Pallavi SK, Shashidhara LS. Signaling interactions between squamous and columnar epithelia of the Drosophila wing disc. Journal of cell science. 2005;118: 3363–70. doi: 10.1242/jcs.02464 16079280

[6] Schittny JC. Development of the lung. Cell and Tissue Research. 2017;367: 427–444. doi: 10.1007/s00441-016-2545-0 28144783 PMC5320013

[7] Rackley CR, Stripp BR. Building and maintaining the epithelium of the lung. Journal of Clinical Investigation. 2012;122: 2724–2730. doi: 10.1172/JCI60519 22850882 PMC3408736

[8] Rognoni E, Watt FM. Skin Cell Heterogeneity in Development, Wound Healing, and Cancer. Trends in Cell Biology. 2018;28: 709–722. doi: 10.1016/j.tcb.2018.05.002 29807713 PMC6098245

[9] Hayashi S, Kondo T. Development and Function of the Drosophila Tracheal System. Genetics. 2018;209: 367–380. doi: 10.1534/genetics.117.300167 29844090 PMC5972413

[10] Pope KL, Harris TJC. Control of cell flattening and junctional remodeling during squamous epithelial morphogenesis in Drosophila. Development. 2008;135: 2227–2238. doi: 10.1242/dev.019802 18508861

[11] Lowe JS, Anderson PG. Epithelial Cells. Stevens Lowes Human Histology. Elsevier; 2015. pp. 37–54. doi: 10.1016/B978-0-7234-3502-0.00003–6

[12] Jockusch BM, Bubeck P, Giehl K, Kroemker M, Moschner J, Rothkegel M, et al. The molecular architecture of focal adhesions. Annual review of cell and developmental biology. 1995;11: 379–416. doi: 10.1146/annurev.cb.11.110195.002115 8689563

[13] Hynes RO. Integrins: versatility, modulation, and signaling in cell adhesion. Cell. 1992;69: 11–25. doi: 10.1016/0092-8674(92)90115-s 1555235

[14] Spradling AC. Developmental genetics of oogenesis. *The Development of Drosophila Melanogaster*. 1993. pp. 1–70. Available: http://www.cshlpress.org/default.tpl?cart=1422971378323094563&fromlink=T&linkaction=full&linksortby=oop_title&—eqSKUdatarq=850

[15] Wu X, Tanwar PS, Raftery LA. Drosophila follicle cells: Morphogenesis in an eggshell. Seminars in Cell and Developmental Biology. 2008. pp. 271–282. doi: 10.1016/j.semcdb.2008.01.004 18304845 PMC2430523

[16] Bastock R, St Johnston D. Drosophila oogenesis. Current Biology. 2008;18: R1082–R1087. doi: 10.1016/j.cub.2008.09.011 19081037

[17] Brigaud I, Duteyrat J-L, Chlasta J, Le Bail S, Couderc J-L, Grammont M. Transforming Growth Factor /activin signalling induces epithelial cell flattening during Drosophila oogenesis. Biology Open. 2015;4: 345–354. doi: 10.1242/bio.201410785 25681395 PMC4359740

[18] Grammont M. Adherens junction remodeling by the Notch pathway in Drosophila melanogaster oogenesis. Journal of Cell Biology. 2007;177: 139–150. doi: 10.1083/jcb.200609079 17420294 PMC2064118

[19] Xi R, McGregor JR, Harrison DA. A gradient of JAK pathway activity patterns the anterior-posterior axis of the follicular epithelium. Developmental Cell. 2003. doi: 10.1016/s1534-5807(02)00412-4 12586061

[20] González-Reyes A, St Johnston D. Patterning of the follicle cell epithelium along the anterior-posterior axis during Drosophila oogenesis. Development (Cambridge, England). 1998;125: 2837–46. Available: http://www.ncbi.nlm.nih.gov/pubmed/9655806 9655806 10.1242/dev.125.15.2837

[21] Tworoger M, Larkin MK, Bryant Z, Ruohola-Baker H. Mosaic analysis in the drosophila ovary reveals a common hedgehog-inducible precursor stage for stalk and polar cells. Genetics. 1999;151: 739–48. Available: http://www.ncbi.nlm.nih.gov/pubmed/9927465 9927465 10.1093/genetics/151.2.739 PMC1460513

[22] Assa-Kunik E, Torres IL, Schejter ED, Johnston DS, Shilo B-Z. Drosophila follicle cells are patterned by multiple levels of Notch signaling and antagonism between the Notch and JAK/STAT pathways. Development. 2007;134: 1161–1169. doi: 10.1242/dev.02800 17332535

[23] Melani M, Simpson KJ, Brugge JS, Montell D. Regulation of Cell Adhesion and Collective Cell Migration by Hindsight and Its Human Homolog RREB1. Current Biology. 2008;18: 532–537. doi: 10.1016/j.cub.2008.03.024 18394891

[24] Sahu A, Ghosh R, Deshpande G, Prasad M. A Gap Junction Protein, Inx2, Modulates Calcium Flux to Specify Border Cell Fate during Drosophila oogenesis. PLOS Genetics. 2017;13: e1006542. doi: 10.1371/journal.pgen.1006542 28114410 PMC5256874

[25] Twombly V, Blackman RK, Jin H, Graff JM, Padgett RW, Gelbart WM. The TGF-beta signaling pathway is essential for Drosophila oogenesis. Development (Cambridge, England). 1996;122: 1555–1565. 8625842 10.1242/dev.122.5.1555

[26] Tsuneizumi K, Nakayama T, Kamoshida Y, Kornberg TB, Christian JL, Tabata T. Daughters against dpp modulates dpp organizing activity in Drosophila wing development. Nature. 1997;389: 627–631. doi: 10.1038/39362 9335506

[27] Inoue H, Imamura T, Ishidou Y, Takase M, Udagawa Y, Oka Y, et al. Interplay of signal mediators of decapentaplegic (Dpp): molecular characterization of mothers against dpp, Medea, and daughters against dpp. Molecular biology of the cell. 1998;9: 2145–2156. doi: 10.1091/mbc.9.8.2145 9693372 PMC25468

[28] Hamaratoglu F, Affolter M, Pyrowolakis G. Dpp / BMP signaling in flies: From molecules to biology. Seminars in Cell and Developmental Biology. 2014;32: 128–136. doi: 10.1016/j.semcdb.2014.04.036 24813173

[29] Muzzopappa M. Multiple roles of the F-box protein Slimb in Drosophila egg chamber development. Development. 2005;132: 2561–2571. doi: 10.1242/dev.01839 15857915

[30] S R, FS N-S, G B, T S. cornichon and the EGF receptor signaling process are necessary for both anterior-posterior and dorsal-ventral pattern formation in Drosophila. Cell. 1995;81: 967–978. doi: 10.1016/0092-8674(95)90016-0 7540118

[31] Zacharioudaki E, Bray SJ. Tools and methods for studying Notch signaling in Drosophila melanogaster. Methods. 2014;68: 173–182. doi: 10.1016/j.ymeth.2014.03.029 24704358 PMC4059942

[32] Spéder P, Brand AH. Gap junction proteins in the blood-brain barrier control nutrient-dependent reactivation of Drosophila neural stem cells. Developmental Cell. 2014;30: 309–321. doi: 10.1016/j.devcel.2014.05.021 25065772 PMC4139190

[33] Nakagawa S, Maeda S, Tsukihara T. Structural and functional studies of gap junction channels. Current Opinion in Structural Biology. 2010;20: 423–430. doi: 10.1016/j.sbi.2010.05.003 20542681

[34] Miao G, Godt D, Montell DJ. Integration of Migratory Cells into a New Site In Vivo Requires Channel-Independent Functions of Innexins on Microtubules. Developmental Cell. 2020;54: 501–515.e9. doi: 10.1016/j.devcel.2020.06.024 32668209 PMC7484434

[35] Orai C, Peinelt C, Vig M, Koomoa DL, Beck A, Nadler MJS, et al. Amplification of CRAC current by STIM1 and. nature cell biology. 2006;8: 6–11. doi: 10.1038/ncb1435 16733527 PMC5685802

[36] Soboloff J, Spassova MA, Tang XD, Hewavitharana T, Xu W, Gill DL. Orai1 and STIM reconstitute store-operated calcium channel function. Journal of Biological Chemistry. 2006;281: 20661–20665. doi: 10.1074/jbc.C600126200 16766533

[37] Bach EA, Ekas LA, Ayala-Camargo A, Flaherty MS, Lee H, Perrimon N, et al. GFP reporters detect the activation of the Drosophila JAK/STAT pathway in vivo. Gene Expression Patterns. 2007;7: 323–331. doi: 10.1016/j.modgep.2006.08.003 17008134

[38] Sinha A, Khadilkar RJ, Vinay KS, Sinha AR, Inamdar MS. Conserved regulation of the JAK/STAT pathway by the endosomal protein asrij maintains stem cell potency. Cell Reports. 2013;4: 649–658. doi: 10.1016/j.celrep.2013.07.029 23972987 PMC4673900

[39] Bertet C, Rauzi M, Lecuit T. Repression of Wasp by JAK/STAT signalling inhibits medial actomyosin network assembly and apical cell constriction in intercalating epithelial cells. Development. 2009;136: 4199–4212. doi: 10.1242/dev.040402 19934015

[40] Jordan P, Karess R. Myosin light chain-activating phosphorylation sites are required for oogenesis in Drosophila. Journal of Cell Biology. 1997;139: 1805–1819. doi: 10.1083/jcb.139.7.1805 9412474 PMC2132636

[41] Majumder P, Aranjuez G, Amick J, McDonald JA. Par-1 controls myosin-II activity through myosin phosphatase to regulate border cell migration. Current Biology. 2012;22: 363–372. doi: 10.1016/j.cub.2012.01.037 22326025 PMC3298626

[42] Kim YS, Fritz JL, Seneviratne AK, VanBerkum MFA. Constitutively active Myosin Light Chain Kinase alters axon guidance decisions in Drosophila embryos. Developmental Biology. 2002. doi: 10.1006/dbio.2002.0768 12221012

[43] Harris TJC, Tepass U. Adherens junctions: From molecules to morphogenesis. Nature Reviews Molecular Cell Biology. 2010. doi: 10.1038/nrm2927 20571587

[44] Oda H, Uemura T, Harada Y, Iwai Y, Takeichi M. A Drosophila Homolog of Cadherin Associated with Armadillo and Essential for Embryonic Cell-Cell Adhesion. Developmental Biology. 1994;165: 716–726. doi: 10.1006/dbio.1994.1287 7958432

[45] Wang Y, Riechmann V. The Role of the Actomyosin Cytoskeleton in Coordination of Tissue Growth during Drosophila Oogenesis. Current Biology. 2007. doi: 10.1016/j.cub.2007.06.067 17656094

[46] Vachias C, Fritsch C, Pouchin P, Bardot O, Mirouse V. Tight coordination of growth and differentiation between germline and soma provides robustness for Drosophila egg development. Cell Reports. 2014. doi: 10.1016/j.celrep.2014.09.035 25373901

[47] Mukai M, Kato H, Hira S, Nakamura K, Kita H, Kobayashi S. Innexin2 gap junctions in somatic support cells are required for cyst formation and for egg chamber formation in Drosophila. Mechanisms of Development. 2011;128: 510–523. doi: 10.1016/j.mod.2011.09.005 22001874

[48] Bohrmann J, Zimmermann J. Gap junctions in the ovary of Drosophila melanogaster: localization of innexins 1, 2, 3 and 4 and evidence for intercellular communication via innexin-2 containing channels. BMC Developmental Biology. 2008;8: 111. doi: 10.1186/1471-213X-8-111 19038051 PMC2631599

[49] Bauer R, Lehmann C, Fuss B, Eckardt F, Hoch M. The Drosophila gap junction channel gene innexin 2 controls foregut development in response to Wingless signalling. Journal of cell science. 2002;115: 1859–67. Available: http://www.ncbi.nlm.nih.gov/pubmed/11956317 11956317 10.1242/jcs.115.9.1859

[50] Giuliani F, Giuliani G, Bauer R, Rabouille C. Innexin 3, a New Gene Required for Dorsal Closure in Drosophila Embryo. Fanning AS, editor. PLoS ONE. 2013;8: e69212. doi: 10.1371/journal.pone.0069212 23894431 PMC3722180

[51] Smendziuk CM, Messenberg A, Vogl AW, Tanentzapf G. Bi-directional gap junction-mediated soma-germline communication is essential for spermatogenesis. Development (Cambridge, England). 2015;142: 2598–609. doi: 10.1242/dev.123448 26116660 PMC6514411

[52] Lehmann C, Lechner H, Löer B, Knieps M, Herrmann S, Famulok M, et al. Heteromerization of Innexin Gap Junction Proteins Regulates Epithelial Tissue Organization in Drosophila. Molecular Biology of the Cell. 2006;17: 1676–1685. doi: 10.1091/mbc.e05-11-1059 16436513 PMC1415333

[53] Gomez JM, Wang Y, Riechmann V. Tao controls epithelial morphogenesis by promoting Fasciclin 2 endocytosis. The Journal of cell biology. 2012;199: 1131–43. doi: 10.1083/jcb.201207150 23266957 PMC3529531

[54] Liu S, Zhang H, Duan E. Epidermal Development in Mammals: Key Regulators, Signals from Beneath, and Stem Cells. International Journal of Molecular Sciences. 2013;14: 10869–10895. doi: 10.3390/ijms140610869 23708093 PMC3709707

[55] Arita K, Akiyama M, Tsuji Y, McMillan JR, Eady RAJ, Shimizu H. Changes in gap junction distribution and connexin expression pattern during human fetal skin development. Journal of Histochemistry and Cytochemistry. 2002;50: 1493–1500. doi: 10.1177/002215540205001109 12417615

[56] Koster MI, Roop DR. Mechanisms Regulating Epithelial Stratification. Annual Review of Cell and Developmental Biology. 2007;23: 93–113. doi: 10.1146/annurev.cellbio.23.090506.123357 17489688

[57] Tepass U, Gruszynski-DeFeo E, Haag TA, Omatyar L, Torok T, Hartenstein V. shotgun encodes Drosophila E-cadherin and is preferentially required during cell rearrangement in the neurectoderm and other morphogenetically active epithelia. Genes & Development. 1996;10: 672–685. doi: 10.1101/gad.10.6.672 8598295

[58] Felix M, Chayengia M, Ghosh R, Sharma A, Prasad M. Pak3 regulates apical-basal polarity in migrating border cells during Drosophila oogenesis. Development. 2015;142: 3692–3703. doi: 10.1242/dev.125682 26395489

