# Supplementary figures and images for: Germline soma communication mediated by gap junction proteins regulates epithelial morphogenesis
Source: PLoS Genet. 2021 Aug 3;17(8):e1009685. doi: 10.1371/journal.pgen.1009685 (PMC8330916; doi:10.1371/journal.pgen.1009685)

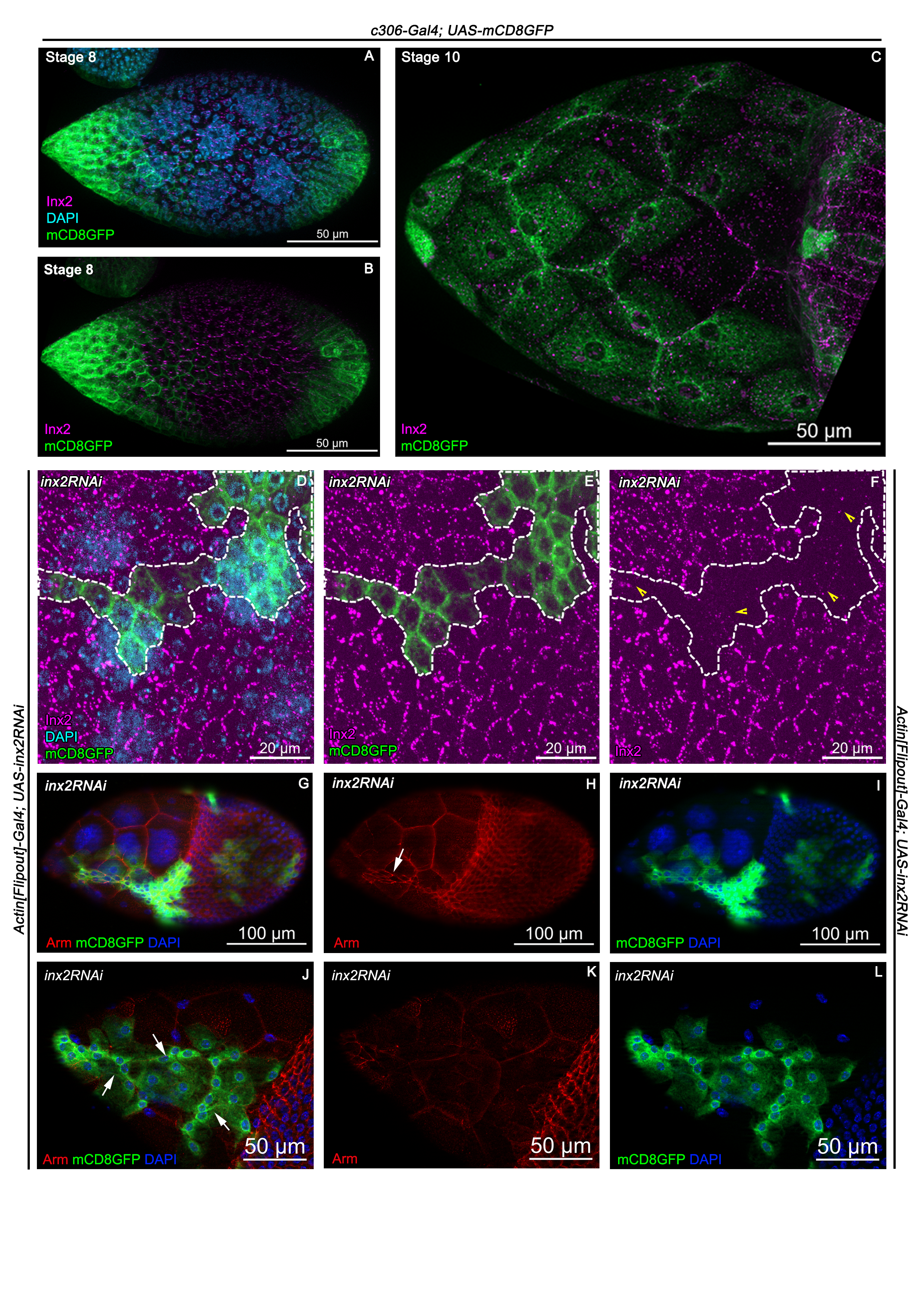

Supplement: S1 Fig — (A-C) Egg chambers of indicated stages depicting the expression pattern of c306-Gal4. GFP is in Green, Inx2 in Magenta and DAPI in Blue in A. Please note expression of c306-Gal4 in the anterior follicle cells. (D-L) Clonal over expression of inx2RNAi mediated by actin[Flipout] -Gal4. Clones are marked in Green. (D-F) inx2RNAi overexpressing clone outlined with a dotted line. Inx2 is in Magenta. DAPI is Blue in D. Please note reduction in the Inx2 level in the RNAi expressing clones in F. Yellow arrow head mark the residual Inx2 protein in the clone. (G-L). Armadillo is in Red and DAPI is in Blue. Arrows point to unstretched cells in H. (J) Small sets of clones at the anterior end (Arrows) exhibiting stretching defect. (J-L) Please note moderate phenotype where follicle cells (arrows) have lost their cuboidal shape but are unable to stretch. (TIF) [file pgen.1009685.s001.tif]

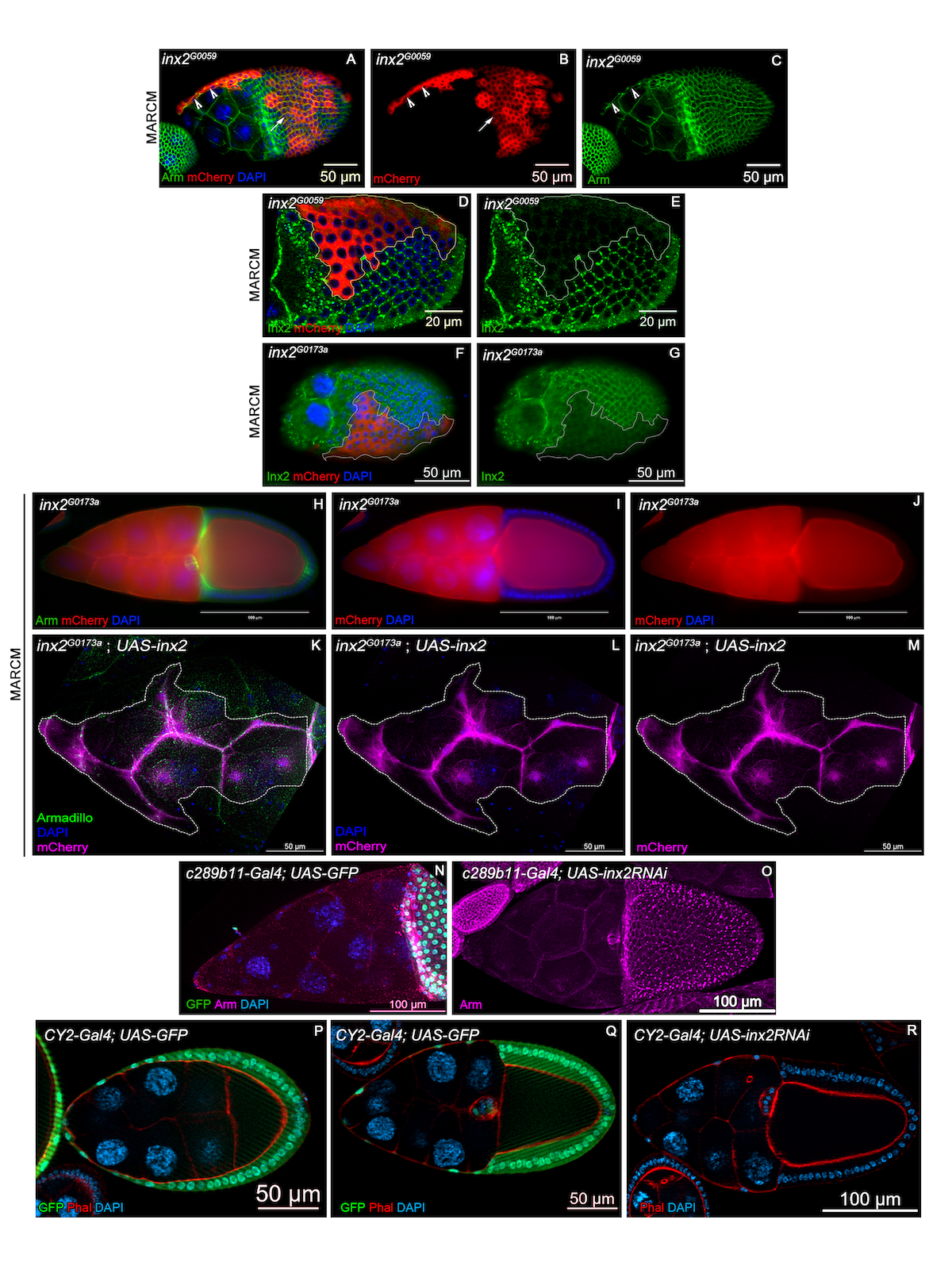

Supplement: S2 Fig — (A-M) MARCM analysis with inx2 mutants. (A-E) mCherry in Red marks the inx2G0059 mutant clones. Arm is Green in A-C and DAPI is Blue in A and D. Arrowhead marks the unstretched follicle cells. Arrow points to the posterior clones, which appear to be normal in A & B. (D-G) inx2 mutant alleles are near nulls (protein). Please note strong down regulation of Inx2 protein in inx2 mutant posterior clones inx2G0059 (D-E) and inx2G073a (F-G). The clones are highlighted in dotted outline, marked by presence of mcherry in Red and Inx2 is in Green. (H-J) Stage 10 egg chambers with inx2G073a germline clones of Inx2 marked in Red, DAPI in Blue and Arm in Green. Please note the egg chambers have normal morphology. (K-M) Surface view of inx2G073a AFCs rescued by overexpression of Inx2 cDNA. mcherry is in Magenta marking the inx2G073a AFCs. Arm in Green and DAPI in Blue. (N-R) Egg chambers of indicated genotypes. (N-O) Armadillo in Magenta in N & O, GFP in green denotes the expression domain of c289b11-Gal4 in N and cy2-Gal4 in P & Q. and DAPI in blue. (O & R) Please note that depletion of Inx2 function in the main body follicle cells in O or in centripetal cells in R doesn’t impede shape change of anterior follicle cells. Phalloidin is Red, in P, Q and R. Please note that depletion of Inx2 function in the main body follicle cells doesn’t impede shape change of anterior follicle cells. (TIFF) [file pgen.1009685.s002.tiff]

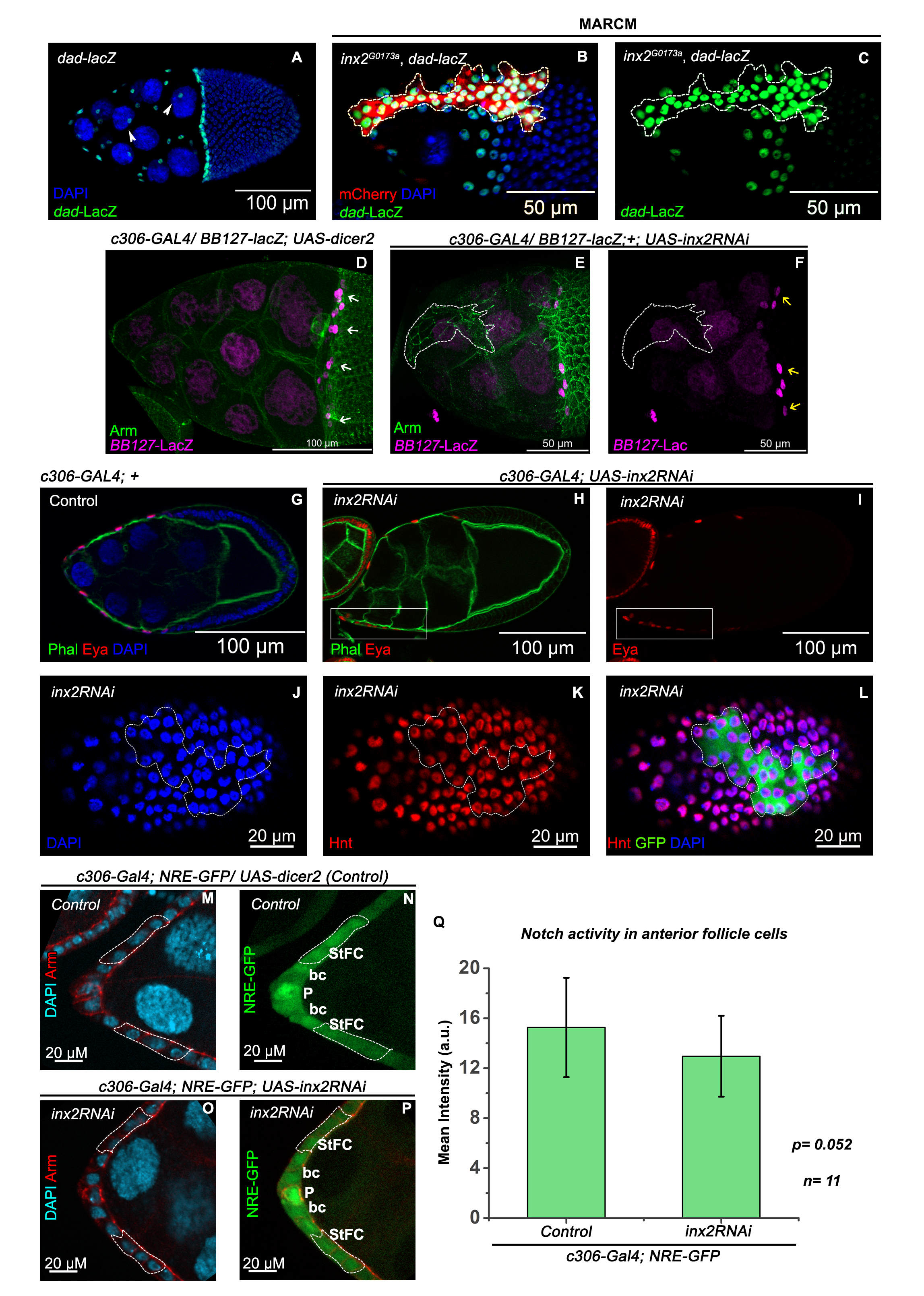

Supplement: S3 Fig — (A-C) Stage 10 egg chambers of indicated genotypes. dad-lacz and DAPI imaged in Green and Blue respectively. Note that dad-lacz marks the squamous and centripetal follicle cells. Arrowhead mark the squamous fate (B-C) MARCM analysis. Stage 10 egg chambers harboring inx2G0173a mutant follicle cells marked in Red by mcherry and highlighted by dotted lines. The unstretched anterior follicle cells express dad-lacz. (D-E) Egg chambers of Indicated genotypes. BB127-lacz in Magenta and Arm in Green. White dotted outline marks the unstretched AFCs in E and F. Arrow in D & F marks the BB127-lacz expression in the centripetal cells. Please note absence of BB127-lacz expression in the unstretched AFCs (dotted outline). (G-I) Transcription factor Eya is excluded from the main body follicle cells. Over expression of inx2RNAi by c306-Gal4 exhibit defect in shape transition in AFCs (boxed in H & I). Red is Eya, Green is Phalloidin and DAPI in blue. Please note Eya is expressed in the AFCs that exhibit stretching defect. (J-Q) Inx2 functions independent of Notch to mediate the stretching of the anterior follicle cells. (J-L) Clonal over expression of inx2RNAi mediated by actin [Flipout] Gal4 in stage 8 egg chamber. Clonal area is outlined and marked in Green. Hindsight is in Red and DAPI is in Blue. Please note that there is no difference in the level of Hindsight protein between the clones and non-clone follicle cells. (M-P) Stage 8 egg chambers with white dotted line outlining the AFCs that would stretch to acquire squamous fate. Control is c306-Gal4/ UAS-dicer2; NRE-GFP and inx2RNAi (c306-Gal4; NRE-GFP/ UAS-inx2RNAi). NRE-GFP expression is in Green, Arm in Red and DAPI in blue. (Q) Quantification of the NRE-GFP of control and inx2RNAi. ‘n’ stands for the number of egg chambers analyzed. Error bars indicate standard deviation. p is the level of significance. (TIFF) [file pgen.1009685.s003.tiff]

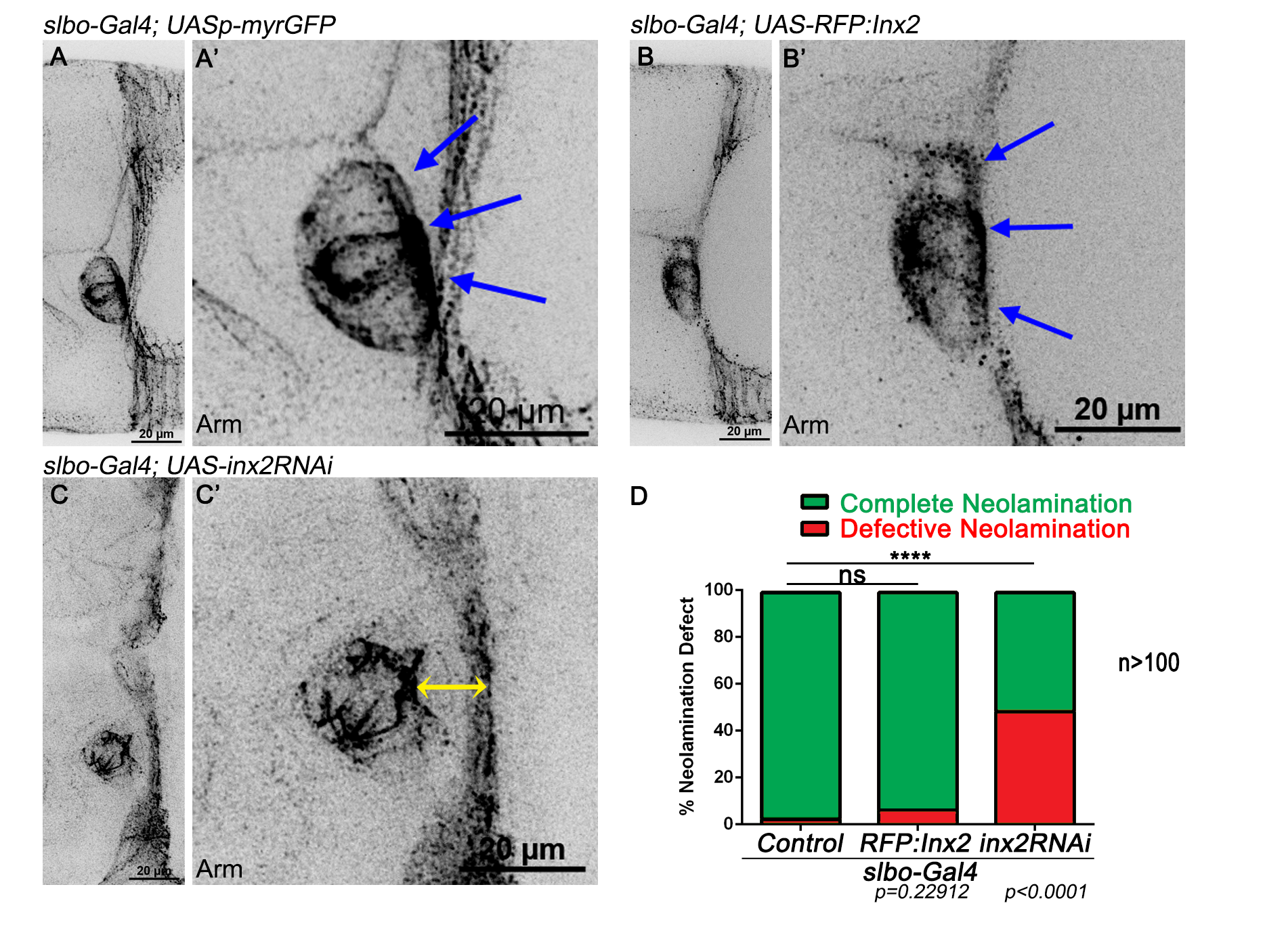

Supplement: S4 Fig — (A-C) images of Border cell cluster near the oocyte boundary. A’. B’ and C’ are the magnified image of A, B and C respectively. Arm is in Black. Blue Arrows in A’ and B’ indicate the neolaminating border cell clusters. Double end yellow arrow in C’ marks the border cells that exhibit neolamination defect. (D) Quantification of Neolamination Defect in the indicated genotypes. Please note that unlike inx2RNAi, overexpression of RFP:Inx2 in border cells clusters exhibited normal neolamination. ‘n’ stands for the number of egg chambers analyzed. p is the level of significance. ns stands for not significant. (TIFF) [file pgen.1009685.s004.tiff]

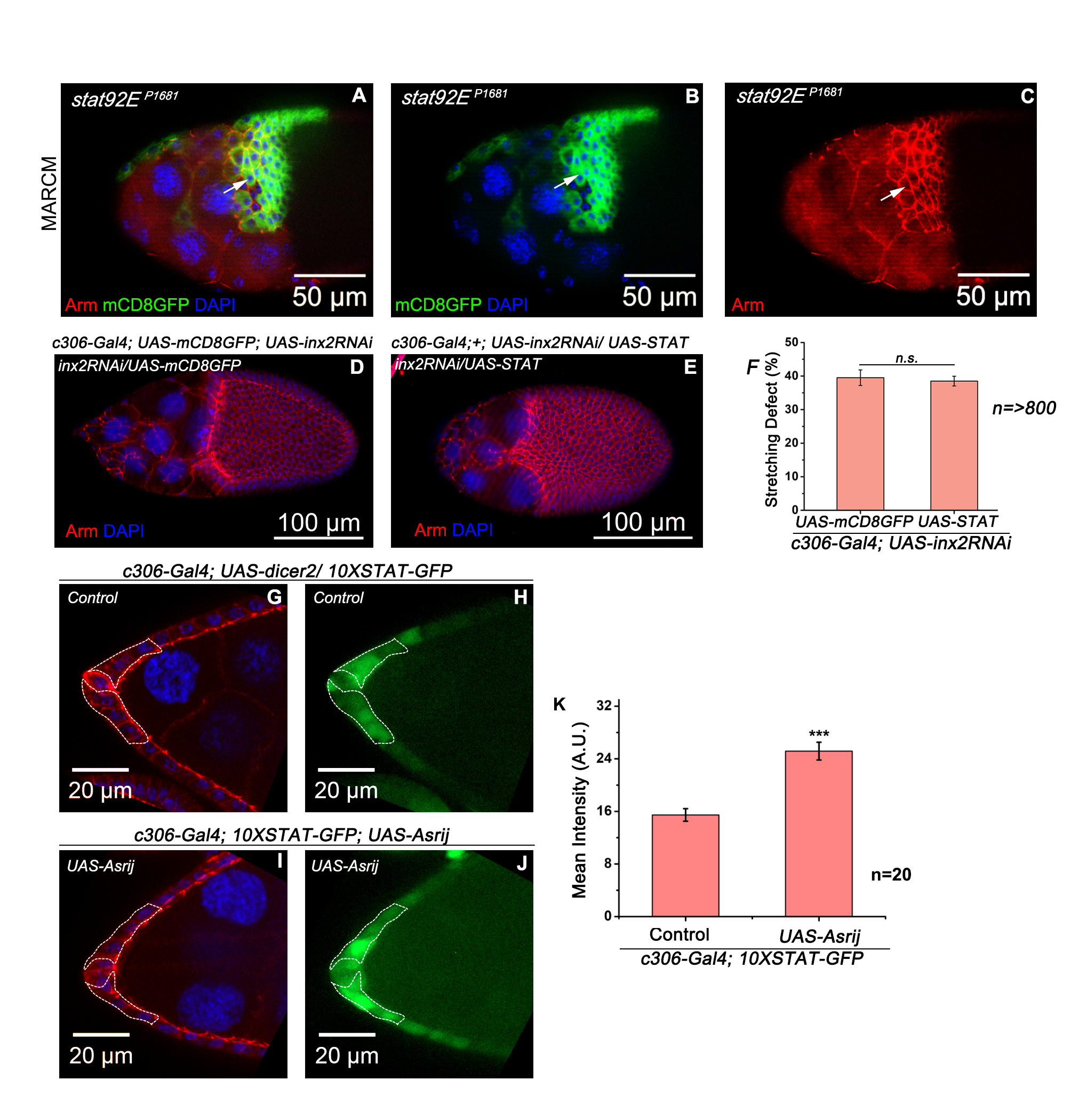

Supplement: S5 Fig — MARCM analysis. (A-C) stat92EP1681 mutant clones are marked in Green, Armadillo in Red and DAPI in blue. Arrow marks the unstretched follicle cells in the mutant clones. (D-F) Overexpression of STAT fails to rescue the Inx2RNAi induced phenotype in AFCs. Quantification of stretching defects in the stage 10 egg chambers of the indicated genotypes. “n” is the number of egg chambers evaluated. Please note that there is no significant difference (ns) in the percentage of observed phenotype for the two genotypes in D and F. (G-K) Overexpression of Asrij activates JAK-STAT signaling in the follicle cells. (G-J) Stage 8 egg chambers of the indicated genotypes. 10XSTAT-GFP expression is in Green in H & J DAPI is Blue. in G and I. (K) Quantification of the 10XSTAT-GFP. Please note increase in the levels of 10XSTAT-GFP in the follicle cells overexpressing Asrij. ‘n’ indicates the number of egg chambers analyzed. *** represent p-value <0.001. Error bars represent Standard Error of Mean. (TIFF) [file pgen.1009685.s005.tiff]

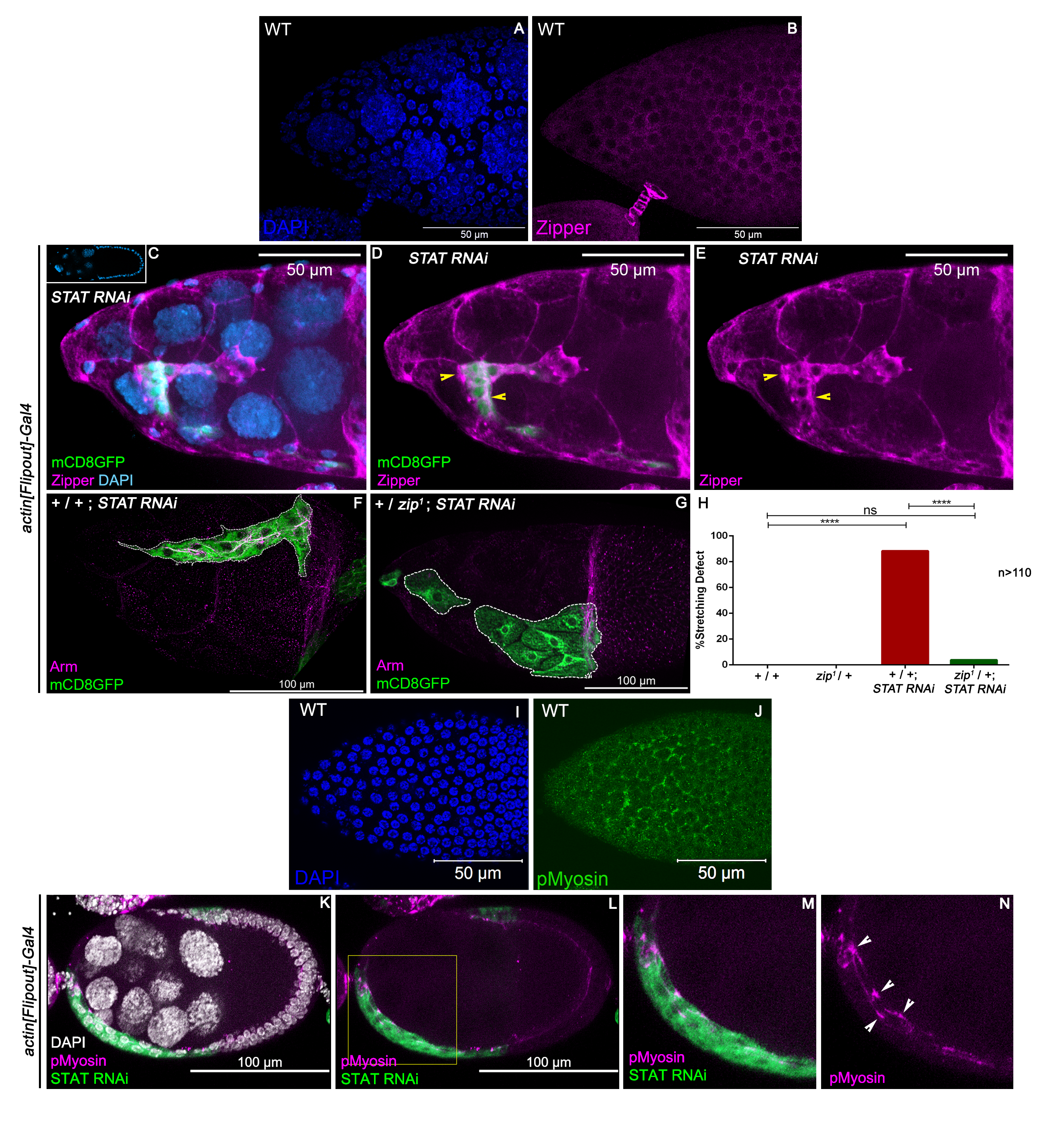

Supplement: S6 Fig — (A-B) AFCs of the Wild type egg chambers. (A) DAPI in Blue. (B) Zipper expression is in Magenta. Please note that Zipper levels are lower in the follicle cells. High level of Zipper is detected in the stalk cells. (C-G) Mosaic analysis-employing actin [Flipout] Gal4 resulting in overexpression of statRNAi in clones. The clones are marked in Green. (C-E) Representative image of the whole egg chamber exhibiting statRNAi induced stretching defect Zipper is in Magenta and DAPI in Blue. Inset in C is the representative image of the corresponding egg chamber. Please note Zipper enrichment in cells exhibiting stretching defect in D & E (marked by yellow arrow head). The border cell cluster in the center exhibits high level of Zipper staining in C to E. (F-H) Rescue of stretching defect of STAT depleted follicle cells when Zip levels (zip1/+) are reduced. statRNAi overexpressing clones outlined in Green. Please note the rescue in cell size of STAT depleted AFCs in G. (H) Quantification of Stretching defect for the indicated genotypes. **** represents p-value <0.0001. n stands for the number of egg chamber analyzed. ‘ns’ stands for not significant. (I-J) Phospho-myosin in the wildtype follicle cells. Please note transient accumulation of Phospho-myosin in the cells undergoing shape change. (K-N) Representative image of the whole egg chamber exhibiting statRNAi induced stretching defect. (K-N) pMyosin in Magenta. M and N are magnified inset of the rectangular outline in L. White Arrowheads in N marks retention of pMyosin in follicle cells exhibiting stretching defect. (TIFF) [file pgen.1009685.s006.tiff]

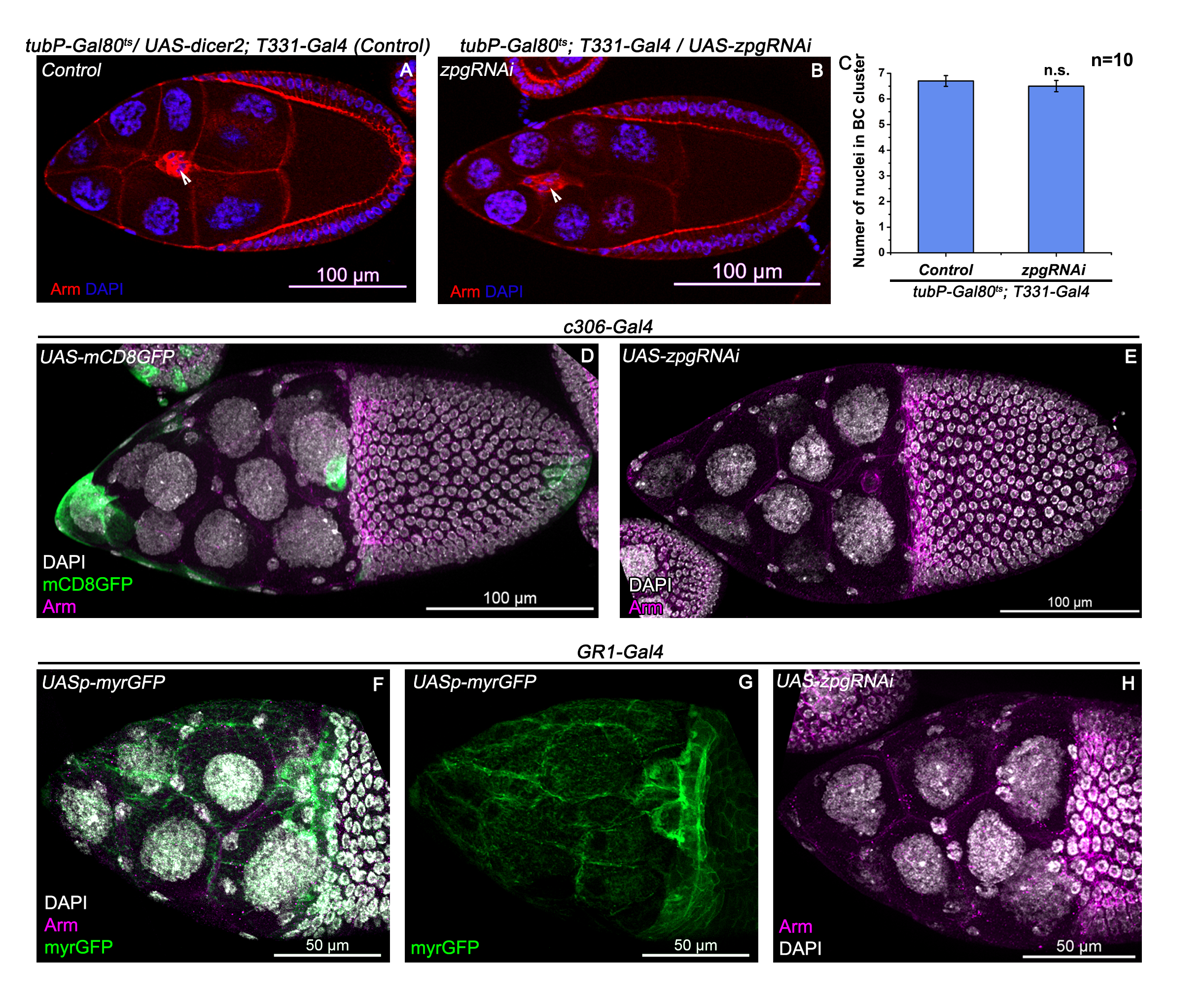

Supplement: S7 Fig — (A-B) Egg chambers of indicated genotypes. Armadillo is in Red and DAPI in Blue. (C) Quantification of number of border cells in migrating clusters of control (A) and zpgRNAi (B). ‘ns’ indicates statistically insignificant. ‘n’ stands for number of clusters analyzed. (D-H) Down regulation of Zpg function in the follicle cells doesn’t affect shape transition of cuboidal cells to squamous fate. Stage 10 egg chambers of indicated genotypes. GFP is in Green in D, F and G. DAPI is in Grey and Arm in Magenta. (TIFF) [file pgen.1009685.s007.tiff]
