# Supplementary material for: Germline soma communication mediated by gap junction proteins regulates epithelial morphogenesis
Source: PLoS Genet. 2021 Aug 3;17(8):e1009685. doi: 10.1371/journal.pgen.1009685 (PMC8330916; doi:10.1371/journal.pgen.1009685)
Supplement: S1 Table — (PDF) [file pgen.1009685.s013.pdf]

Fig 1

A-F	*c306-GAL4/+; UAS-mCD8GFP/+; +/+*
G-L	*c306-GAL4/+; +/+; UAS-inx2RNAi/+*
M-O'	*hsFLP, FRT19A tubP-Gal80/ FRT19A inx2G0173a;+; actin-Gal4/UAS-mCherry*
P-R	*hsFLP, FRT19A tubP-Gal80/ FRT19A inx2G0173a; UAS-inx2; actin-Gal4/UAS-mCherry*

Fig 2

A-C	*hsFLP/ + ; UAS-RFP:Inx2/ +; Actin[Flipout]-Gal4*
D-F	*hsFLP/ + ; UAS-RFP:Inx2/ +; Actin[Flipout]-Gal4*
G	*c306-GAL4/+; UAS-mCD8GFP/+; UAS-MoesinGFP/+*
H	*c306-GAL4/+; UAS-mCD8GFP/+; UAS-inx2RNAi/+*
I	*c306-GAL4/+; +/+; UAS-inx2RNAi/UAS-Orai*
J	*c306-GAL4/+; +/+; UAS-inx2RNAi/UAS-itpr*

Fig 3

A	*c306-GAL4/+; UAS-mCD8GFP/+; +/+*
B	*c306-Gal4/+; +/+; UAS-inx2RNAi/+*
C	*c306-Gal4/hop27; +/+; UAS-inx2RNAi/+*
D	*c306-GAL4/+; +/+; UAS-inx2RNAi/STAT92EP1681*
F-H	*hsFLP, FRT19A tubP-Gal80, FRT19A hop27;+; actin-Gal4/UAS-mCherry*
I-J	*c306-GAL4/UAS-dicer2; 10X STAT GFP/UAS-tdTomato; +/+*
K-L	*c306-GAL4/+; 10X STAT GFP/ UAS-tdTomato; UAS-inx2RNAi/+*
N	*c306-GAL4/+; UAS-mCD8GFP/+; UAS-inx2RNAi/+*
O	*c306-GAL4/+; +; UAS-inx2RNAi/ UAS-Asrij*

Fig 4

A-C	*hsFLP/+; +; Actin<Flipout>GAL4, UAS-mCD8GFP/ UAS-inx2RNAi*
D	*c306-GAL4/+; +/+; UAS-inx2RNAi/+*
E	*c306-GAL4/+; +/+; UAS-inx2RNAi/WASp3*

Fig 5

A-B	*hsFLP /+; +/ +; actin<Flipout>GAL4, UAS-mCD8GFP/ UAS-inx2RNAi*
D-E	*hsFLP, FRT19A tubP-Gal80/ FRT19A inx2G0173a; +/ +; actin-Gal4/UAS-mCherry*
F-G	*hsFLP/+; UAS-MLCK.CA/+; Actin<Flipout>GAL4, UAS-mCD8GFP*
H-I	*hsFLP/+; +/ +; Actin<Flipout>GAL4, UAS-mCD8GFP/ UAS-sqhRNAi*

Fig 6

A-B	*CsWT*
C-D	*hsFLP, FRT19A tubP-Gal80/ FRT19A inx2G0173a;+; actin-Gal4/UAS-mCherry*
E	*c306-Gal4/+; +/+; UAS-inx2RNAi/+*
F	*c306-Gal4/+; shg6115/+; UAS-inx2RNAi/+*
G	*c306-Gal4/+; shgp34-1/+; UAS-inx2RNAi/+*

Fig 7

A-C	*CsWT*
D-F	*hsFLP, FRT19A tubP-Gal80/ FRT19A inx2G0173a;+; actin-Gal4/UAS-mCherry*
G-H	*c306 -Gal4/+; UAS-RFP:Inx2/+;+/+*
I-K	*hsFLP, FRT19A tubP-Gal80/ FRT19A inx2G0173a; UAS-RFP:Inx2/ +; GR1-Gal4/+*
L	*c306-Gal4/+; +/+; UAS-inx2RNAi/+*
M	*c306-Gal4/+; +/+; UAS-inx2RNAi/zpg2533*
N	*c306-Gal4/+; +/+; UAS-inx2RNAi/zpg5352*

Fig 8

A^1-B^5	*c306-GAL4/UAS-dicer2; UAS-GCaMP6m/+; T331-GAL4/+*
C^1-C^5	*c306-GAL4/+; UAS-GCaMP6m/+; T331-GAL4/UAS-zpgRNAi*
F-F''	*UAS-dicer2/+; tubP-Gal80ts/+, T331-GAL4/+*
G-G''	*.+/+; tubP-Gal80ts/+, T331-GAL4/UAS-zpgRNAi*

S1

A-C	*c306-Gal4/+;+; UAS-mCDGFP/+*
D-L	*hsFLP/+;+/ +; actin<Flipout>-Gal4, UAS-mCD8GFP / UAS-inx2RNAi*

S2

A-E	*hsFLP, FRT19A tubP-Gal80/ FRT19A inx2G0059;+/+; actin-Gal4/ UAS-mcherry*
F-J	*hsFLP, FRT19A tubP-Gal80/FRT19A inx2G0173a;+/+; actin-Gal4/ UAS-mcherry*
K-M	*hsFLP, FRT19A tubP-Gal80/FRT19A inx2G0173a; UAS-Inx2/+; actin-Gal4/ UAS-mcherry*
N	*+/+;+/+; c289b11-Gal4/UAS-GFP*
O	*+/+;+/+; c289b11-Gal4/ UAS-inx2RNAi*
P-Q	*+/+; CY2-Gal4/+; UAS-GFP/+*
R	*+/+; CY2-Gal4/+; UAS-inx2RNAi/+*

S3

A	*Cs WT*
B-C	*hsFLP, FRT19A tubP-Gal80/FRT19A inx2[G0173a];+; actin-Gal4, UAS-mcherry/ dad-lacZ*
D	*c306-Gal4/ BB127-lacZ; UAS-Dicer2/+;+/+*
E-F	*c306-Gal4/ BB127-lacZ; +/+; UAS-inx2RNAi/+*
G	*c306-Gal4/+;+/+;+/+*
H-I	*c306-Gal4/+;+/+; UAS-inx2RNAi/+*
J-L	*hsFLP/+;+/+; actin<Flipout>-Gal4, UAS-mCD8GFP/ UAS-inx2RNAi*
M-N	*c306-Gal4/+; NRE-GFP/ UAS-Dicer2;+/+*
O-P	*c306-Gal4/+; NRE-GFP/+; UAS-inx2RNAi/+*

S4

A	*+/+; slbo-Gal4/UASp-myrGFP; +/+*
B	*+/+; slbo-Gal4/ UAS-RFP:Inx; +/+*
C	*+/+; slbo-Gal4/+;UAS-inx2RNAi/+*

S5

A-C	*hsFLP/+; actin-Gal4, UAS-mCD8GFP/ +; FRT82B tubP-Gal80/ FRT82B* $stat92E^{P1681}$
D	*c306-Gal4/+; UAS-mCD8GFP/+; UAS-inx2RNAi/+*
E	*c306-Gal4/+;+/+; UAS-inx2RNAi/ UAS-STAT*
G-H	*c306-Gal4/+; UAS-Dicer2/ 10XSTAT-GFP;+/+*
I-J	*c306-Gal4/+; 10XSTAT-GFP/+; UAS-Asrij/+*

S6

A-B	*Cs WT*
C-E	*hsFLP/+;+/+; actin<Flipout>-Gal4, UAS-mCD8GFP/ UAS-STAT RNAi*
F	*hsFLP/+;+/+; actin<Flipout>-Gal4, UAS-mCD8GFP/ UAS-STAT RNAi*
G	*hsFLP/+; zip1/+; actin<Flipout>-Gal4, UAS-mCD8GFP/ UAS-STAT RNAi*
I-J	*Cs WT*
K-N	*hsFLP/+;+/+; actin<Flipout>-Gal4, UAS-mCD8GFP/ UAS-STAT RNAi*

S7

A	*+/+; tubP-Gal80[ts]/UAS-dicer2;T331-Gal4/ +*
B	*+/+; tubP-Gal80[ts]/+;T331-Gal4/ UAS-zpg RNAi V22*
D	*c306-Gal4/+;+/+; UAS-mCD8GFP/+*

E	*c306-Gal4/+;+/+; UAS-zpg RNAi V10/+*
F-G	*+/+;+/UASp-myrGFP; GR1-Gal4/ +*
H	*+/+;+/+; GR1-Gal4/ UAS-zpg RNAi V10/+*
